# Thermal niche helps to explain the ability of dung beetles to exploit disturbed habitats

**DOI:** 10.1038/s41598-020-70284-8

**Published:** 2020-08-07

**Authors:** Victoria C. Giménez Gómez, José R. Verdú, Gustavo A. Zurita

**Affiliations:** 1 Instituto de Biología Subtropical, Universidad Nacional de Misiones-CONICET, Bertoni 85, 3370 Puerto Iguazú, Misiones Argentina; 2 Instituto Universitario de Investigación – Centro Iberoamericano de la Biodiversidad, Universidad de Alicante, Alicante, Spain; 3 Facultad de Ciencias Forestales, Universidad Nacional de Misiones-CONICET, El Dorado, Misiones Argentina

**Keywords:** Ecology, Physiology

## Abstract

In terrestrial ecosystems, insects face a wide range of temperatures among habitats and time; consequently, the thermal niche is one of the main determinants of habitat selection and temporal patterns of activity. The replacement of native forests changes micro-climatic conditions and reduces the diversity of dung beetles; however, the physiological mechanisms behind these changes are not clear. We explore the role of the thermal niche in dung beetles to explain the ability of native species to exploit human-created habitats. Using infrared thermography, we measured variables associated with the thermal niche in 17 native species and used linear mixed-effects model and ANOVAs to compare disturbed habitats and the native forest. Endothermy and body mass explained the ability of dung beetles to exploit human-created open habitats. Small and diurnal species with very low endothermy were able to exploit deforested open habitats; evening/nocturnal/crepuscular species showed similar body mass and high endothermy in all habitats. Regarding thermoregulation mechanisms, none of the species (except one) showed defined or efficient mechanisms of physiological thermoregulation. In view of the accelerated process of forest replacement and climate change, a more profound understanding of the physiological requirements of species is essential to predict and mitigate future extinctions.

## Introduction

At local scales, anthropogenic habitat disturbance usually modifies microclimatic conditions (e.g. forest canopy loss), resulting in novel thermal challenges for organisms (e.g. open habitats)^1,2^. In tropical and subtropical forests, recent studies have shown a marked reduction in the abundance and taxonomic and functional diversity of dung beetles in land uses with complete loss of canopy cover, such as pastures devoted to cattle raising^2–4^. In contrast, land uses preserving canopy cover (such as tree plantations and silvopastoral systems) can partially preserve the structure of the dung beetle assemblages of the native forest^2,5–7^. Differences in dung beetle diversity among land uses differing in canopy cover have been associated mainly with a marked increase in ground level temperature^2,8–10^ and with the low tolerance of forest dung beetles to high temperatures (typical of open habitats during the day)^3,11–13^. Although previous studies have suggested that the physiological intolerance of forest dung beetles to high temperatures is a potential constraint to exploit disturbed habitats^2,3,14^, this hypothesis has never been tested. This knowledge gap on dung beetle thermal biology led us to ask the following question: can the thermal niche of dung beetles explain the occupation of disturbed habitats, and taxonomic and functional changes reported in previous studies? We tested this central question in the southern Atlantic forest of South America, one of the most diverse and threatened ecosystems worldwide^15–17^.

The physiological responses of insects, especially those associated with temperature (endothermy, thermoregulation, thermal tolerance), are some of the main determinants of their spatial^18,19^ and temporal distribution^20,21^. Temperature controls several biochemical^22,23^ and physiological functions, behavior, and development in insects^1,24^. In terrestrial ecosystems, insects face a wide variety of climatic conditions (e.g. temperature, humidity) among different habitats and throughout the day and year^25^. Consequently, the thermal components of the ecological niche (thermal niche) partly influence the use of habitat and the daily and seasonal patterns of the activity of individuals^18,26–28^.

In heterothermic flying insects, the thermal niche is defined by the ability to raise body temperature above environmental temperature (defined as excess temperature or endothermy) and the ability to regulate the internal temperature within an optimal range of temperatures (defined as thermoregulation)^28^. Endothermy, or excess temperature, can be measured as the difference between body and environmental temperature^18,25,28–30^, and this difference is affected primarily by flight muscle activity prior to take-off and during flight^28,29,31^. Regarding thermoregulation, individuals can regulate their temperature through behavior (e.g. posterior leg posture that affects turbulence convective cooling during the flight)^31^, morphology (e.g. color, size) and/or physiology (e.g. hemolymph circulation, respiration)^1,28^. Both the endothermy and thermoregulation of insects have received much attention due to their central role in their ecology (competition for a resource and/or establishment of reproductive pairs), evolution^28,32^, distribution, and response to climatic changes^28,33,34^. In dung beetles, two physiological mechanisms of thermoregulation have been described: the *abdominal active heat transfer* (AAHT) (typical of diurnal species) and the *abdominal passive heat transfer* (APHT) (mainly found in nocturnal species)^31^. In the AAHT, individuals are able to transfer the excess of heat from the thorax to the abdomen through the movement of the hemolymph driven by the pumping of the abdomen, to be later partially eliminated from the abdomen through the tegument by convection and conduction, or through the spiracles during respiration by evaporation and convection^31,35^. In the APHT, individuals retain the heat in the thorax by using anatomical adaptations, such as thoracic hairs and air sacs, preventing heat from being transferred to the abdomen^31^. Although these mechanisms, as well as endothermy, have been used to explain patterns of temporal^20,31^ and spatial distribution in dung beetles^18,19,36^, they have never been used to explain the response of insect populations and communities to anthropogenic disturbances. It is important to highlight that endothermy is one component of thermoregulation; therefore endothermy and thermoregulation are not independent variables. However, since in this study we independently evaluated and discussed both processes, we considered them separately.

In this context, the main objective of this study was to explore the role of the thermal niche to explain (1) the ability of some native forest species to exploit human-created open habitats and (2) changes in taxonomic and functional diversity following forest replacement. Based on differences in ground level temperatures among habitats preserving canopy cover (native forests and agroforestry parklands) and habitats without canopy cover (open pastures) (Table S1)^5^, we expected: (1) higher endothermy in diurnal species found only in habitats with canopy cover and lower endothermy in those found in habitats without canopy cover; (2) similar endothermy among evening/nocturnal/crepuscular species (E/N/C species), irrespectively of their preferred habitat; and (3) AAHT in diurnal species and APHT in E/N/C species, irrespectively of their preferred habitat. Additionally, we explored other variables associated with flight (and therefore with endothermy and thermoregulation) that could explain differences in habitat use: temperature changes of the thorax and abdomen during prolonged flight (defined as thorax and abdomen temperature slopes), the difference between both slopes and the temperature required to initiate continuous flight in laboratory conditions, here referred to as “minimum thoracic tethered flapping temperature” (this terms is equivalent to “minimum thoracic take-off temperature” under field conditions). All physiological variables used in this study were selected over other variables (e.g. CTmax, ULT) because they are directly associated with the thermal niche, and therefore measured within the normal conditions for a particular species. We also explored the effect of body mass in species response, given that the thorax temperature, the minimum thoracic tethered flapping temperature and endothermy are mass dependent in individuals with a mass below 2 g^18,37,38^.

## Methods

### Study area and environments sampled

We conducted the field work in one of the largest remnants of the semideciduous Atlantic forest of Argentina (between 25° 58′ 21″ S, 54° 17′ 22″ W and 26° 36′ 32″ S, 54° 41′ 43″ W), a threatened ecosystem (Supplementary Figure S1). The region is located in the northeast of Argentina, and is characterized by a seasonal warm climate, with annual temperatures between 17 and 22 °C and an average annual rainfall of 2,000 mm, without a dry season^39^. On a landscape scale, the study area includes continuous native forest in protected areas (“Parque Nacional Iguazú”, “Parque Provincial Urugua-í”, “Parque Provincial Península”, “Reserva Privada de Fundación Vida Silvestre”, among others), large tracts of exotic tree monocultures (mainly of *Pinus taeda* Linnaeus) for the production of cellulose and wood, and small properties dedicated to subsistence agriculture with corn (*Zea mays* Linnaeus), tobacco (*Nicotiana tabacum* Linnaeus), and yerba mate (*Ilex paraguariensis* Augustin Saint-Hilaire) and small pastures for cattle raising^40,41^.

**Figure 1:**
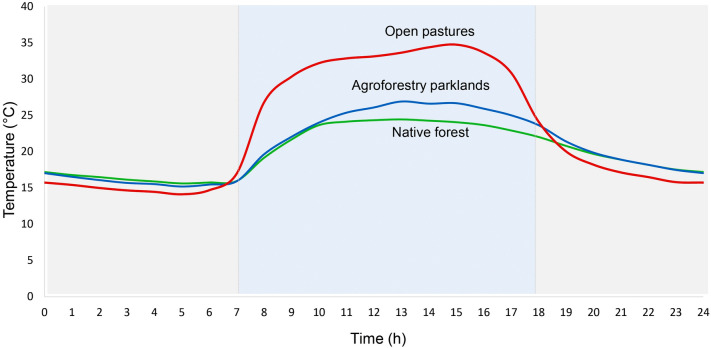
Mean temperature values measured during 12 full days in three habitats of the Atlantic forest of Argentina: native forest (green line), agroforestry parklands (blue line) and open pastures (red line)^5^. The full day (24 h) was divided in two periods based on changes in temperature and the time of sunrise and sunset in spring: (1) from 7:00 to 18:00 h (light blue period) and (2) from 18:00 to 7:00 h (gray period).

Within the study area, we collected dung beetles in three habitats: protected native forests, agroforestry parklands with cattle, and open pastures for cattle raising (Supplementary Figure S1). The native forest is a heterogeneous habitat formed by three to six arboreal strata and an understory composed of ferns, bamboos and shrubs^42,43^, in which the soil is primarily composed of sand with high contents of organic carbon^7^. Agroforestry parklands are remnants of native forest dedicated to livestock raising, where the vegetation is composed of native trees, lianas and an understory of shrubs, herbs and bamboos^2^, and in which the soil is characterized by high clay contents^7^ . In contrast to these two environments, open pastures are dominated by exotic grasses planted for livestock consumption and dispersed trees^2^, in which the soil is characterized by high clay content probably because of soil erosion^7^. Within each habitat, we selected three sites (or replicates), separated by a minimum distance of 30 km to increase regional representativeness. In relation to the microclimatic conditions, the native forest and agroforestry parklands show similar temperature at ground level throughout the entire day, whereas open pastures show higher temperature at ground level during the day and similar temperature at ground level during the night^5^ (Supplementary Table S1, Fig. 1). We used automatic temperature and humidity sensors (HOBO Pro) to record temperature at ground level in all sampling sites. We placed the automatic sensor 1 m above the ground and recorded temperature every 5 min during 12 continuous days in spring. From the complete temperature dataset (considering the 12 continuous days), we calculated the mean, maximum and minimum temperature in the day and night.

### Habitat selection and daily activity

We selected species to be analyzed based on the results of two previous studies performed in the same study area^2,5^. According to those results, dung beetles are more abundant in agroforestry parklands, exhibiting intermediate abundance in native forests, and lower abundance in open pastures. In addition, native forests and agroforestry parklands showed a similar number of species, which was higher than in open pastures. Based on these studies, we used the Indicator Value Method (IndVal)^44^ to select representative species (indicator and detector species) of each habitat (native forests, agroforestry parklands and open pastures). The IndVal uses a permutation test to assess the significance of individual indicator and detector species and is calculated through the following equation:

$$ IndVal_{ij } = A_{ij} \times B_{ij} \times 100 $$

where *A*_*ij*_ is a measure of specificity that represents species abundance in a given habitat with respect to the total number of collected species, and *B*_*ij*_ is a measure of fidelity that represents the number of habitats where the species is present with respect to the total number of habitats considered^44–46^. *A* and *B* are multiplied because they represent independent information about species distribution. The indicator values range from 0% (no indicator) to 100% (perfect indicator). Species with significant (*P* < 0.05) results above 70% were considered indicator species whereas species with an intermediate IndVal value, between 45 and 70%, were considered detector species. Indicator species are characteristic of a particular habitat and may decline rapidly under changing ecological conditions. Detector species exhibit a different degree of preference for different habitats and relative changes in their abundance among habitats might indicate the direction in which change is occurring^46^. We performed this analysis using PC-Ord 5^47^. Considering the IndVal results (Supplementary Table S2), we selected three native forest species, eight agroforestry parkland species, and three open pasture species (Table 1).

**Table 1:** Thermal niche of dung beetles inhabiting the native forests and different habitats (Agroforestry parklands and Open pastures) of the Atlantic forest of Argentina.

Habitat	Activity	Species	N	Average mass (g) (± s.d.)	Average endothermy (°C) (± s.d.)	Average thorax slope (± s.d.)	Average abdomen slope (± s.d.)	Average environmental slope (± s.d.)	Slopes difference Tth − Tabd (df = 1)		Slopes difference Tabd − Tenv (df = 1)	
									H	P	H	P
Native forests	D	Canthon smaragdulus (Fabricius, 1781)**	8	0.156 ± 0.08	3.138 ± 0.68	− 0.0025 ± 0.02	− 0.0034 ± 0.01	0.0004 ± 1.7e−3	2.8e−3	0.9807	0.03	0.8561
		Coprophanaeus saphirinus (Sturm, 1826)**	14	0.423 ± 0.23	7.254 ± 1.06	0.0026 ± 0.01	− 0.0049 ± 0.01	0.0013 ± 2.8e−3	4.5	0.0345	3.5	0.0595
	E/N/C	Deltochilum brasiliensis (Castelnau, 1,840)**	8	1.008 ± 0.07	2.337 ± 1.26	− 0.0027 ± 0.01	− 0.0008 ± 0.01	− 0.0005 ± 8.9e−4	0.04	0.8775	0.4	0.5546
		Deltochilum furcatum (Castelnau, 1,840)*	3	0.663 ± 0.05	3.985 ± 0.79	0.0104 ± 0.01	− 0.0022 ± 0.01	0.0013 ± 9.9e−4	2.3	0.2000	0.4	0.7000
		Deltochilum morbillosum (Burmeister, 1848)***	6	0.228 ± 0.02	2.885 ± 0.47	0.0002 ± 3.1e−3	− 0.0006 ± 1.5e−3	− 3.33e−5 ± 7.3e−4	0.03	0.8777	0.8	0.4221
Agroforestry parklands	D	Canthon conformis (Harold, 1868)*	13	0.04 ± 0.01	3.332 ± 0.97	− 0.0058 ± 0.01	− 0.0087 ± 0.01	0.0008 ± 1.6e−3	0.4	0.5049	2.1	0.1438
		Canthon quinquemaculatus (Castelnau, 1840)*	10	0.17 ± 0.02	1.841 ± 0.43	− 0.0063 ± 3.5e−3	− 0.0036 ± 4.2e−3	− 0.0001 ± 7.1e−4	2.1	0.1506	3	0.0819
		Canthon histrio (Lepeletier and Serville, 1828)*	8	0.147 ± 0.02	3.771 ± 0.69	− 0.0051 ± 0.01	0.0034 ± 0.01	0.00001 ± 2.1e−3	2.5	0.1304	2.5	0.1304
	E/N/C	Coprophanaeus cyanescens (Olsoufieff, 1924)*	6	1.713 ± 0.28	9.217 ± 3.99	0.0048 ± 0.01	0.0127 ± 0.03	0.0017 ± 2.1e−3	0.1	0.7835	0.1	0.7565
		Deltochilum aff. komareki (Balthasar, 1939)*	5	0.232 ± 0.05	2.019 ± 0.92	0.0021 ± 0.01	− 0.0020 ± 3.5e−3	0.0006 ± 2.1e−3	0.3	0.6905	1.3	0.3095
		Dichotomius carbonarius (Mannerheim, 1829)***	7	0.525 ± 0.05	4.042 ± 0.68	− 0.0033 ± 3.3e−3	− 0.0011 ± 2.6e−3	0.0067 ± 0.01	1.6	0.2203	1.5	0.2328
		Dichotomius mormon (Ljungh, 1799)*	8	1.021 ± 0.16	5.025 ± 1.68	0.0003 ± 0.01	0.0015 ± 4.8e−3	0.0004 ± 1e−3	0.5	0.5054	0.1	0.7422
		Dichotomius sericeus (Harold, 1867)*	5	0.336 ± 0.08	2.779 ± 2.78	0.0099 ± 0.02	0.0022 ± 0.01	− 0.0007 ± 8.7e−4	0.01	> 0.9999	2.4	0.1508
Open pastures	D	Canthon curvodilatus (Schmidt, 1922)**	4	0.027 ± 2.9e−3	0.472 ± 0.14	− 0.0014 ± 0.01	− 0.0004 ± 0.01	0.0004 ± 8.1e−4	0.3	0.6857	1.3	0.3429
		Canthon podagricus (Harold, 1868)**	11	0.028 ± 3.5e−3	1.142 ± 0.73	0.0009 ± 0.01	0.0018 ± 0.02	0.0001 ± 1.5e−3	0.1	0.7926	0.2	0.6222
	E/N/C	Dichotomius nisus (Olivier, 1789)*	6	0.511 ± 0.02	6.143 ± 1.14	0.0006 ± 1.6e−3	0.0011 ± 3.7e−3	0.0014 ± 2.1e−3	0	> 0.9999	0.3	0.6126
		Ontherus sulcator (Fabricius, 1775)*	6	0.317 ± 0.02	2.248 ± 0.33	0.0020 ± 4.6e−3	0.0019 ± 4.8e−3	− 0.0003 ± 1.8e−3	0.03	0.8777	0.2	0.6667

D = diurnal and E/N/C = evening/nocturnal/crepuscular, and asterisks represent the source used to establish the activity: *Daily activity sampling, **Hernández^48^, ***Hernández et al*.*^49^. The value highlighted in bold represents *P* < 0.05 and H is the statistic of Kruskal–Wallis H test. N = number of replicates (sampling size) by species, s.d. = standard deviation and df = degrees of freedom. In slopes difference, T_th_ = thorax temperature, T_abd_ = abdomen temperature and T_env_ = environment temperature.

Based on field observation of the authors and published articles in the same region^48,49^, we also included three species representative of two environments (*Canthon smaragdulus* and *Deltochilum morbillosum* of native forest, *Ontherus sulcator* of open pastures) (Table 1). These species were not selected using the IndVal, due to the low number of individuals captured probably as a consequence of the pitfall method used. However, field observations indicate that these species are abundant and typical from the mentioned habitats.

To describe daily activity of each species, we performed an ad-hoc sampling during five full days (24 h), between November and February 2015–2016. In each habitat, we established 10 pitfall traps (3 habitats × 10 traps = 30 traps in total), five baited with omnivorous dung and five with carrion (to attract both coprophagous and necrophagous species). We divided the 24-h period in two periods, based on changes in ground level temperature^5^ and the time of sunrise and sunset (data provided by the “Servicio de Hidrografía Naval Argentino”): (1) from 7:00 to 18:00 h and (2) from 18:00 to 7:00 h (Fig. 1). According to the number of captures in each period, we classified species as diurnal or evening/nocturnal/crepuscular (E/N/C) when the number of individuals captures in one of the periods was ≥ 60%. Finally, we used previous studies in the Atlantic forest describing daily activity of dung beetles^48,49^, and field observations carried out by the authors to classify species with a low number of captures. According to those two sources of information, seven were classified as diurnal and 10 as evening/nocturnal/crepuscular (E/N/C) (Supplementary Table S4, Table 1).

### Eco-physiological bioassays

To perform eco-physiological experiments, we collected dung beetles in spring and summer (October to February) between 2015 and 2017, by using pitfall traps baited with human feces or rotten meat with leaf litter inside to reduce the mortality of falling individuals. We placed a plastic cover on the plastic cup with an open triangle of 3–4 cm to allow dung beetles to enter but prevent them from escaping. We kept collected individuals for 12 h in the laboratory at 25 °C before the assays, mainly to minimize stress^50–53^. The acclimation temperature of 25 °C was selected because it simulates the average environmental conditions in which the species are found in the field. During that time, we fed coprophagous species with cow dung and necrophagous species with decomposing fish.

Prior to the experiment, we considered several methodological requirements to assure a common physiological state for all individuals: (a) we selected individuals with pre-flight behavior, which is associated with body warm-up (mainly thoracic temperature), and is determined by wing muscle vibration, foreleg and head muscle flexing exercises, and sometimes, abdominal pumping^28,31,54^; and (b) we considered only mature individuals (of approximately the same age) according to external age-grading methods based on the cuticular deterioration of the tibiae and clypeus and on the hardness of the cuticle^55,56^. It is important to clarify that we performed the experiments at the time of day when species were active, that is, during the day for diurnal species and during the evening, night or crepuscule for E/N/C species.

For endothermy and thermoregulation experiments, we fixed dung beetles using a pin with paraffin from the pronotum and suspended them in the air at a height of 1.5 m. Prior to experiments, we weighed individuals using a precision balance (HXT-501, accuracy = 0.1 g). During the take-off and flight of each individual, we recorded a real-time video from a 0.5 m distance with a thermal infrared camera (FLIR ThermaCam T450 with a resolution of 320 × 240 pixels and a microbolometer Focal Plane Array detector with a spectral range of 7.5–13 µm and a thermal sensitivity of < 30 mK at 30 °C). We corrected the temperature measures by reflection, relative humidity, distance from the object, and emissivity^31,53^. To correct by emissivity, we measured cuticle emissivity at different temperatures (50–80 °C) by using fresh cuticles from each species and an electrical tape as reference (ε = 0.95) (Supplementary Table S3). We performed the measurements on individuals maintaining a continuous flight for at least 40 s, with each individual tested once. Considering the diversity of species used in this study and the experience of the authors, 40 s is the minimum time required to adequately estimate physiological variables. Individuals that did not achieve the minimum time, or that did not fly on the first attempt were not considered for analysis.

### Analysis of endothermy and thermoregulation

**Figure 2:**
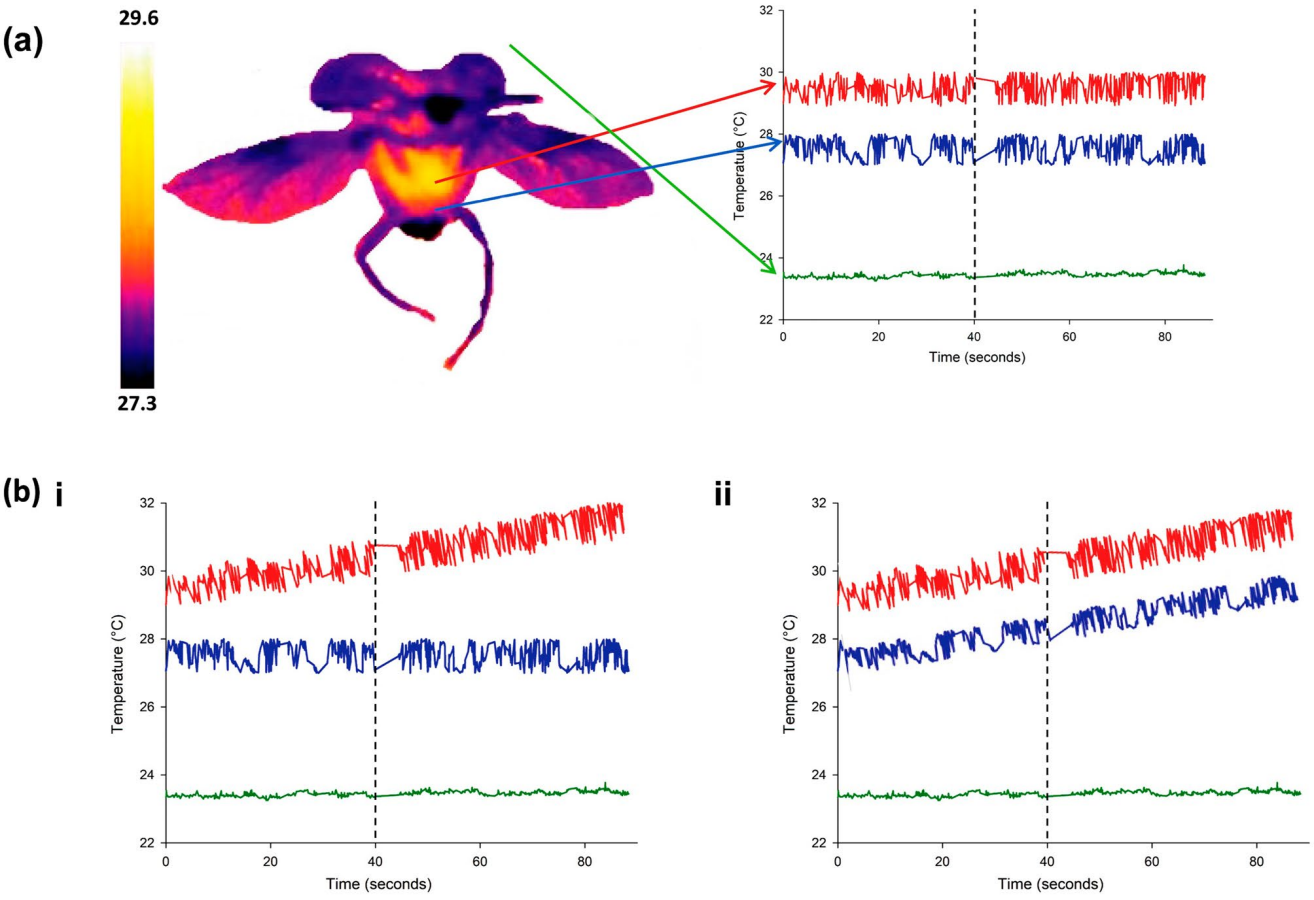
Endothermy and thermoregulation analysis in dung beetles. (**a**) Red circle = thorax temperature (T_th_), blue circle = abdomen temperature (T_abd_) and green circle = environmental temperature (T_env_). During flight, the differences between the mean value of T_th_ and T_env_ represent endothermy (excess temperature) and the difference between the slopes of T_th_ and T_abd_, T_abd_ and T_env_, and their signs represent the physiological thermoregulation mechanisms. The colour scale in the left column and in the insect represents a gradient of body temperature, from high (white and yellow shades) to low (violet and black shades). (**b**) Physiological thermoregulation mechanism: (i) abdominal passive heat transfer (different T_th_ and T_abd_ slopes, positive T_th_ slope, and similar T_abd_ and T_env_ slopes) and (ii) abdominal active heat transfer (similar T_th_ and T_abd_ slopes, positive T_th_ and T_abd_ slopes, different T_abd_ and T_env_ slopes). In all graphs, the vertical dotted line indicates the time at which slopes were considered.

To estimate endothermy and thermoregulation, we selected three areas (3 × 3 pixels each one) during take-off and flight in: (1) the thorax (T_th_) (measured in the metathoracic plate), (2) the abdomen (T_abd_) (measured in the third abdominal sternite) and (3) the nearby environment (T_env_) (Fig. 2). The selected areas correspond to the temperatures used to estimate endothermy and thermoregulation. We measured the temperature from the individuals in lateral view because the elytra would block the top view. Finally, we considered the maximum temperature recorded in each area, because leg movement might cause inaccurate temperature measurements since the thermal camera mistakenly measures leg temperature^31^, and the environmental temperature was recorded on each individual experiment (between 21 and 30.5 °C depending on the species).

We calculated endothermy as the difference between the average value of T_th_ and T_env_ during flight (excess temperature) and estimated the physiological thermoregulation mechanisms from the difference between the temperature changes of the thorax and abdomen (slopes of T_th_ “sT_th_” and T_abd_ “sT_abd_”), and temperature changes of the abdomen and environment (slopes of T_abd_ “sT_abd_” and T_env_ “sT_env_”) during prolonged flight (Fig. 2a). We calculated the sT_th_, and sT_abd_ across time, from take-off to 40 s into continuous flight, for each individual throughout the temperature profiles (FLIR ResearchIR Max + HSDR Version 4.40.8.28 software), and we estimated temperature variation slopes across time from the regression parameters calculated by the least squares method (Statistical Software^57^). In relation to the physiological thermoregulation mechanisms, when sT_th_ and sT_abd_ were different, sT_th_ was positive, sT_abd_ and sT_env_ were similar, the mechanism was called abdominal passive heat transfer (APHT) (Fig. 2b-i); whereas when sT_th_ and sT_abd_ were similar, sT_th_ and sT_abd_ were positive, sT_abd_ and sT_env_ were different, the mechanism was called abdominal active heat transfer (AAHT) (Fig. 2b-ii)^31^.

In addition to endothermy and physiological thermoregulation, we considered the thorax and the abdomen temperature slopes (sT_th_ and sT_abd_), which are additional physiological variables potentially influencing the ability of dung beetles to exploit open areas. Since both temperatures (T_th_ and T_abd_) are sensitive to the heat transferred from the thorax to the abdomen during flight, their slopes can be taken as independent variables because they are not always coupled or correlated (see abdominal passive heat transfer^31^); while both the sT_th_ and sT_abd_ are used in APHT and AAHT patterns to indicate whether there is heat transfer from the thorax to the abdomen during continuous flight, sT_th_ also reflects generated heat and sT_abd_ reflects heat loss^31,37^. We also considered the difference between them (sT_th_ and sT_abd_) as an estimation of the amount of heat transferred from the thorax to the abdomen during flight; a greater difference between these slopes indicates less heat transferred from the thorax to the abdomen^31^. Finally, the analysis included the minimum thoracic tethered flapping temperature, which corresponds to the temperature required to take-off and start the flight. In heterothermic insects, this temperature is directly associated with the ability to raise body temperature above environmental temperature (endothermy) by using their wing muscles^28^ and is independent of the environmental temperature^58^.

### Data analysis

To test the predictions of endothermy and associated variables (T_th_ and T_abd_ slopes, difference between both slopes, and minimum thoracic tethered flapping temperature), we performed a linear mixed-effects model for each variable (five in total). In the model we used daily activity (diurnal and E/N/C) and habitat type (native forests, agroforestry parklands and open pastures) as explanatory variables, the physiological variables (endothermy, T_th_ and T_abd_ slope, difference between them and minimum thoracic tethered flapping temperature) as dependent variables, and species identity as a random variable. Finally, we considered the body mass as a co-variable in the model because endothermy, thorax temperature and minimum thoracic tethered flapping temperature are mass dependent in individuals with a mass below 2 g^36–38^, as is the case of all the individuals of this study. As an example, in the case of endothermy, the full model was:

$$ {\text{Model}} = {\text{lmer }}\left( {{\log}\left( {{\text{endothermy}}} \right)\sim {\text{habitat}}*{\text{activity}} + {\text{mass}} + \left( {1|{\text{species}}} \right)} \right) $$

All variables followed a normal distribution with the only exception of endothermy; in this last case endothermy was log transformed to reach normality. We performed an analysis of variance (ANOVA, type III), with Wald Chi-square test, to test the significance of the global model for each variable and pairwise post-hoc comparisons (Tukey tests). All the analyses were performed using the ‘lme4’, ‘car’ and ‘emmeans’ packages in R^59–61^. Additionally, we performed Kruskal–Wallis H tests and pairwise post-hoc comparisons (Conover tests)^62^ to test the difference in body mass among diurnal and E/N/C species that use different habitats (native forests, agroforestry parklands and open habitats). We performed this analysis to explore whether species body mass varies (a) among different habitats (considering the habitat as an independent variable), (b) within the same habitat (considering the activity as an independent variable); and (c) to associate these results with those of the physiological variables.

To test our predictions for thermoregulation, we compared the pairs of slopes (sT_th_ and sT_abd_/sT_abd_ and sT_env_) for each species using Kruskal–Wallis H tests (INFOSTAT software^63^) and we considered the signs of the thorax and abdomen temperature slopes for the analysis. For positive slopes we assigned a positive sign, for negative slopes the sign was negative.

As we used multiple testing, this may create possible problems in the correct assessments of p-levels. However, we preferred to do not introduce any correction for various reasons. First, p-level adjustments generally lead to a loss of relevant information associated with the reduction of statistical power. In ecological studies, these approaches have been criticized because their results often lead to underestimated ecological patterns^64^. Also, most of our hypotheses were independent, even when using the same data. Finally, we performed correlations between variables used in more than one hypotheses and, in all cases, we obtained low correlations (r = 0.59 between thorax slope and abdomen slope, r = 0.41 between thorax slope and difference between both slopes, r = − 0.49 between abdomen slope and difference between both slopes).

## Results

### Endothermy and body mass

**Figure 3:**
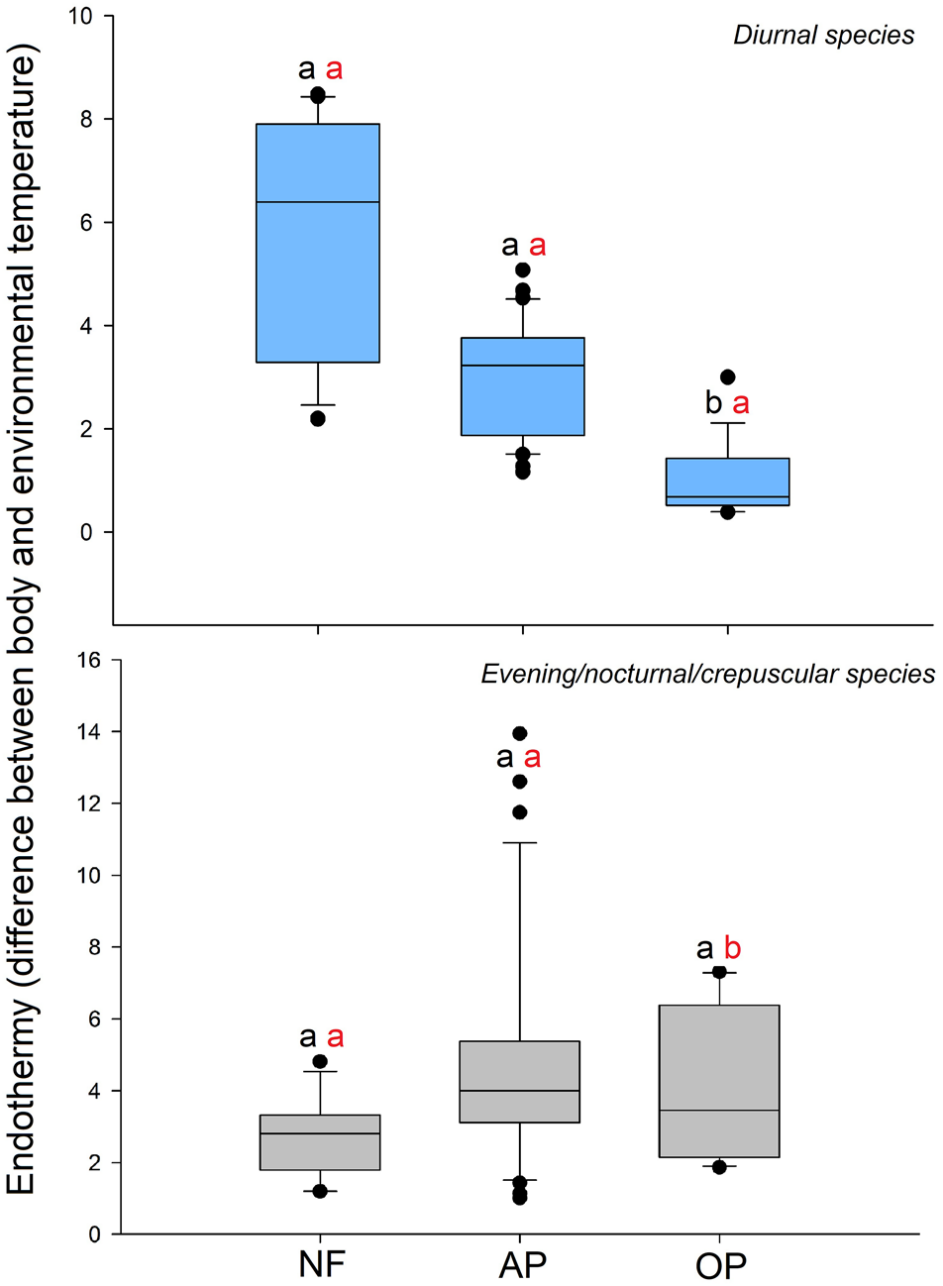
Analysis of variance testing the significance of the linear mixed-effects model for endothermy (whiskers, median and outliers) values among different dung beetles species according to their activity (diurnal = light blue blocks, evening/nocturnal/crepuscular = gray blocks) from the native forests (NF), agroforestry parklands (AP) and open pastures (OP) of the Atlantic forest of Argentina. Common letter are not significantly different (*P* > 0.05, Tukey post-hoc comparison tests). Black letters compare endothermy among species with the same activity and among environments. Red letters = difference in endothermy among species according to their activity (diurnal and evening/nocturnal/crepuscular) in the same environment (e.g. NF).

The most endothermic species was *Coprophanaeus cyanescens* Olsoufieff (9.22 °C over the environmental temperature), whereas the least endothermic one was *Canthon curvodilatus* Schmidt (0.42 °C over the environmental temperature) (Table 1). The analysis of variance testing the significance of the model for endothermy values among different species, while considering habitat type (native forests, agroforestry parklands or open pastures) and daily activity (diurnal or E/N/C), showed a significant interaction between both factors (Table 2). The endothermy of diurnal species from open pastures was lower than that of diurnal species from the native forest and agroforestry parklands (Fig. 3), but showed no differences between diurnal species from the native forest and agroforestry parklands (Table 3). E/N/C species showed similar endothermy in all habitats (Table 3, Fig. 3). Finally, the endothermy between diurnal and E/N/C species differed only in open pastures (Table 4, Fig. 3).

**Table 2:** Results of the analysis of variance testing the significance of the linear mixed-effects model used to compare endothermy, minimum thoracic take-off temperature (T_th_ min of take-off), thorax temperature slope (sT_th_), abdomen temperature slope (sT_abd_) and difference between both (sT_th_–T_abd_) across different habitats (native forests, agroforestry parklands and open pastures) and daily activity (diurnal or evening/nocturnal/crepuscular) in dung beetles from the Atlantic forest of Argentina.

	Endothermy	sT_th_	sT_abd_	sT_th_–sT_abd_	T_th_ min of take-off
Chisq	10.3855	4.2189	1.3649	0.5443	1.7320
*df*	2	2	2	2	2
*P*	**0.005**	0.1213	0.5054	0.7617	0.4206

Species mass was considered as a co-variable and the species identity as a random effect. The value highlighted in bold represents *P* < 0.05. Chisq = statistic, df = degrees of freedom and *P* = *p* value.

**Table 3:** Differences in endothermy, minimum thoracic take-off temperature (T_th_ min of take-off), thorax temperature slope (sT_th_), abdomen temperature slope (sT_abd_) and the difference between both (sT_th_–sT_abd_) across diurnal and evening/nocturnal/crepuscular dung beetle species of different habitats (native forests, agroforestry parklands, open pastures) in the Atlantic forest of Argentina.

		Diurnal					Evening/nocturnal/crepuscular				
		Endothermy	sT_th_	sT_abd_	sT_th_–sT_abd_	T_th min_ of take-off	Endothermy	sT_th_	sT_abd_	sT_th_–sT_abd_	T_th_ min of take-off
Open pastures versus native forests	E t P	1.9616 3.809 **0.0077**	3.78e^−6^ 0.001 1.0000	− 0.0061 − 1.369 0.4051	5.71e^−3^ 1.119 0.5272	2.2690 1.348 0.4005	− 0.2824 − 0.595 0.8256	− 6.71e^−4^ − 0.162 0.9857	− 0.0026 − 0.550 0.8480	2.67e^−3^ 0.529 0.8588	− 0.7321 − 0.470 0.8865
Open pastures versus agroforestry parklands	E t P	1.4264 3.029 **0.0288**	− 6.51e^−3^ − 1.807 0.2342	− 0.0054 − 1.316 0.4265	− 1.26e^−3^ − 0.267 0.9616	0.9877 0.641 0.8011	0.0203 0.047 0.9988	1.10e^-3^ 0.295 0.9532	0.0013 0.294 0.9536	7.97e^−5^ 0.017 0.9998	− 0.4318 − 0.304 0.9506
Native forests versus agroforestry parklands	E t P	0.5352 1.148 0.5075	6.51e^−3^ 2.087 0.1966	− 0.0006 − 0.169 0.9844	6.98e^−3^ 1.595 0.3067	1.2813 0.843 0.6860	− 0.3027 − 0.795 0.7132	− 1.77e^−3^ − 0.530 0.8580	− 0.0038 − 1.008 0.5842	2.59e^3^ 0.634 0.8041	− 0.3003 − 0.240 0.9688

E = estimate (effect size of each contrast), *t* = statistic and *P* = *p* value (Tukey post-hoc comparison tests). The values highlighted in bold represent *P* < 0.05.

**Table 4:** Differences between diurnal and evening/nocturnal/crepuscular dung beetle species in endothermy, minimum thoracic take-off temperature (T_th_ min of take-off), thorax temperature slope (sT_th_), abdomen temperature slope (sT_abd_) and the difference between both (sT_th_–sT_abd_) in three habitats of the Atlantic forest of Argentina: native forests, agroforestry parklands and open pastures.

		Endothermy	sT_th_	sT_abd_	sT_th_–sT_abd_	T_th_ min of take-off
Native forests	E	0.5332	0.0001	− 0.0033	0.0022	− 0.3162
	t	1.133	0.039	− 0.805	0.475	− 0.205
	P	0.2816	0.9702	0.4433	0.6455	0.8411
Agroforestry parklands	E	− 0.3047	− 0.0081	− 0.0065	− 0.0021	− 1.8978
	t	− 0.812	− 2.883	− 2.005	− 0.581	− 1.548
	P	0.4345	**0.0196**	0.0775	0.5750	0.1509
Open pastures	E	− 1.7108	− 0.0005	0.0002	− 0.0008	− 3.3173
	t	− 3.298	− 0.123	0.039	− 0.149	− 1.951
	P	**0.0070**	0.9042	0.9695	0.8842	0.0766

E = estimate (effect size of each contrast), t = statistic and *P* = *p* value (Tukey post-hoc comparison tests). The values highlighted in bold represent *P* < 0.05.

**Figure 4:**
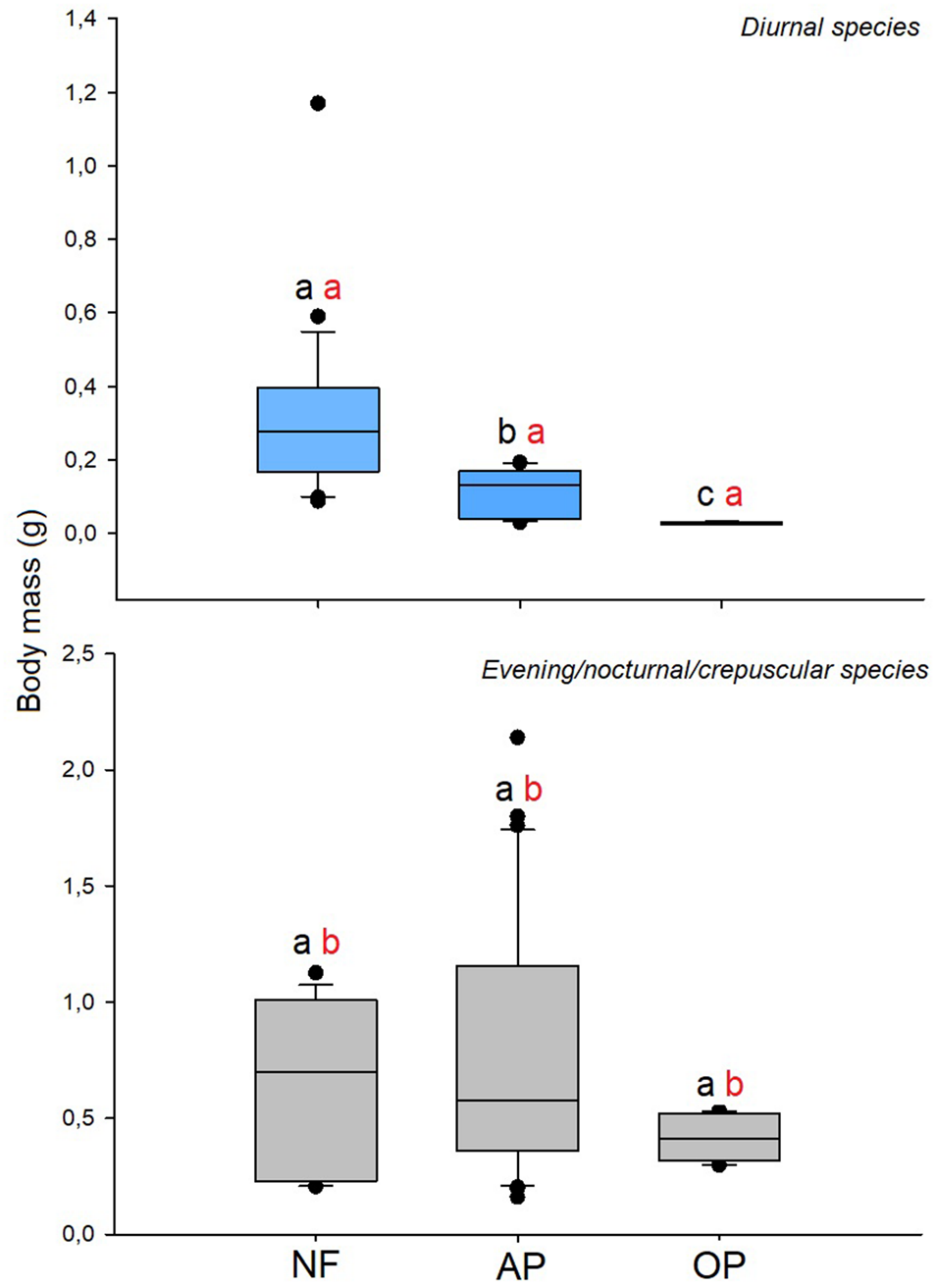
Body mass (whiskers, median and outliers) in diurnal (light blue blocks) and evening/nocturnal/crepuscular (gray blocks) dung beetles from the native forests (NF), agroforestry parklands (AP) and open pastures (OP) of the Atlantic forest of Argentina. Common letters are not significantly different with *P* > 0.05 (Conover post-hoc comparison tests, Kruskal–Wallis H tests). Black letters compare endothermy among species with the same activity and among environments. Red letters = difference in endothermy among species according to their activity (diurnal and evening/nocturnal/crepuscular) in the same environment (e.g. NF).

In relation to body mass, the Kruskal–Wallis H test and post-hoc comparison showed differences among diurnal species and habitat types (H = 45.36; *P* < 0.0001; *df* = 2); species body mass decreased from native forests to open habitats; whereas agroforestry parklands showed an intermediate situation. The body mass of E/N/C species was similar in all habitats (H = 3.95; *P* = 0.1385; *df* = 2) (Fig. 4). Regarding differences in body mass between diurnal and E/N/C species of the same habitat, in all habitats diurnal species were smaller than E/N/C species (native forests H = 5.53; *P* = 0.0187; *df* = 1/agroforestry parklands H = 43.88; *P* < 0.0001; *df* = 1/open pastures H = 19.29; *P* < 0.0001; *df* = 1).

### Thermoregulation mechanisms

**Figure 5:**
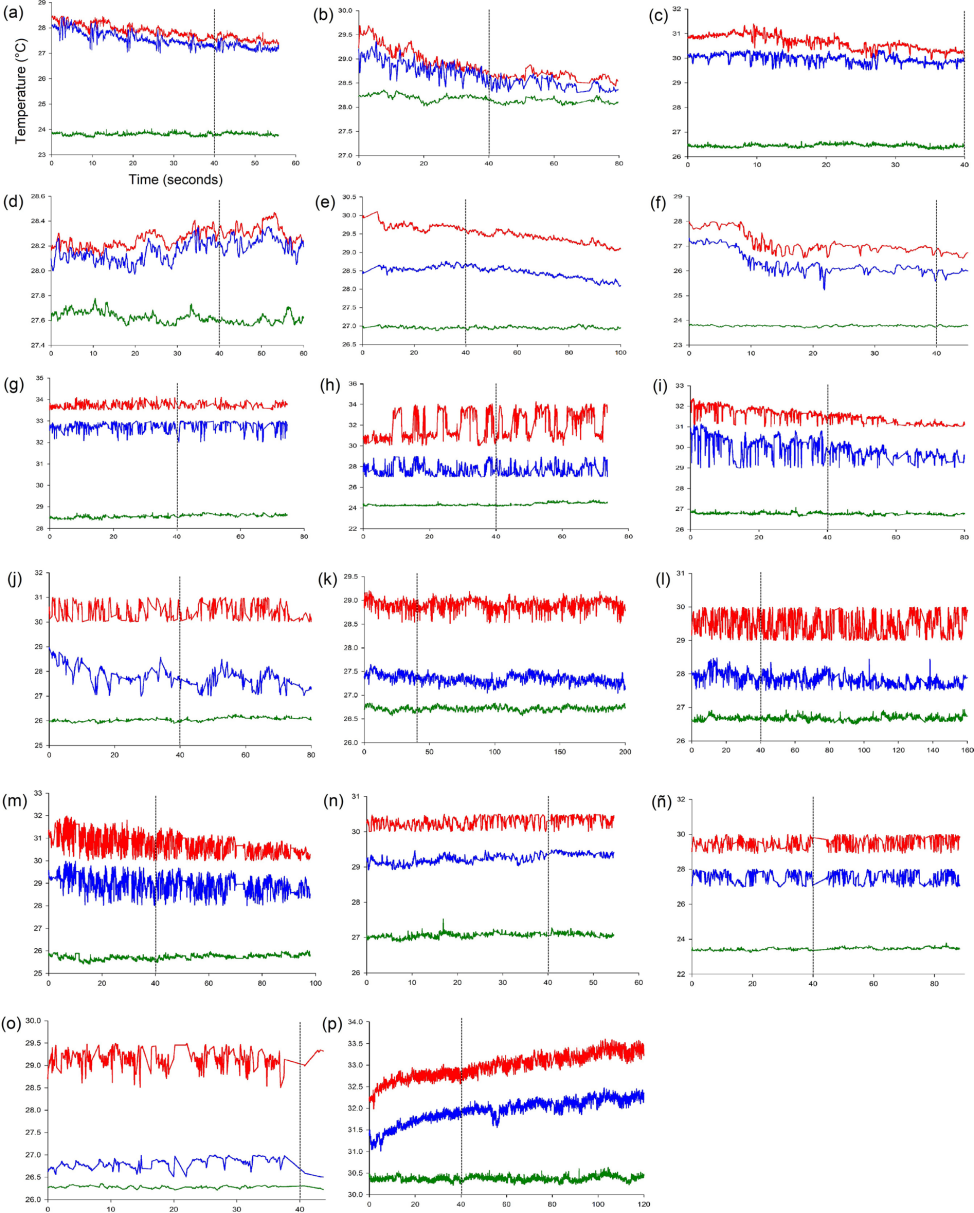
Results of flight thermoregulation experiments in dung beetle species (Kruskal–Wallis H test). Red line = thorax temperature (T_th_), blue line = abdomen temperature (T_abd_) and green line = environmental temperature (T_env_). Each graph shows, as an example, an individual from each of the following species: (**a**) *Canthon conformis*, (**b**) *Canthon curvodilatus*, (**c**) *Canthon histrio*, (**d**) *Canthon podagricus*, (**e**) *Canthon quinquemaculatus*, (**f**) *Canthon smaragdulus*, (**g**) *Coprophanaeus cyanescens*, (**h**) *Coprophanaeus saphirinus*, (**i**) *Deltochilum brasiliensis*, (**j**) *Deltochilum furcatum*, (**k**) *Deltochilum komareki*, (**l**) *Deltochilum morbillosum*, (**m**) *Dichotomius carbonarius*, (**n**) *Dichotomius mormon*, (**ñ**) *Dichotomius nisus*, (**o**) *Dichotomius sericeus* and (**p**) *Ontherus sulcator*. In all graphs, Y axe = Temperature (°C), X axe = Time (seconds) (as it is represented in the graph **a**), and the vertical dotted line indicates the time at which slopes were considered.

Thermoregulation patterns results are shown in Fig. 5. According to the definition of physiological thermoregulation mechanisms (AAHT and APHT) and Kruskal–Wallis H test performed to compare pairs of slopes, only one species (*Coprophanaeus saphirinus* Sturm) showed APHT; this means that sT_th_ and sT_abd_ were different, sT_th_ was positive and sT_abd_ and sT_env_ were similar (Table 1, Fig. 5h). Only some individuals of *Canthon podagricus* Harold, *Coprophanaeus cyanescens* and *Ontherus sulcator* Fabricius presented AAHT: sT_th_ and sT_abd_ were similar and slightly positive, and sT_abd_ and sT_env_ were also similar (Table 1, Fig. 5d, g, p). The remaining species, regardless of their activity, showed no defined physiological thermoregulation mechanisms (neither AAHT nor APHT): sT_th_ and sT_abd_ were similar and negative, whereas sT_abd_ and sT_env_ were also similar (e.g.* Canthon smaragdulus* Fabricius and *Deltochilum brasiliensis* Castelnau) (Table 1, Fig. 5f, i).

Finally, in relation to other variables associated with endothermy and thermoregulation (sT_th_ and sT_abd_, difference between them and minimum thoracic tethered flapping temperature), the analysis of variance testing the significance of the model for these variables was not significant for habitats or daily activity (Table 2).

## Discussion

Recent studies in the Atlantic forest (and in neotropical forests in general) have shown that land uses with complete loss of the canopy cover show a marked reduction in dung beetle abundance and diversity^2,3,5,65^ and that those preserving the canopy cover (at least partially) are often used by native forest dung beetles^2,3,5,7,66,67^. While these differences have been explained by the hypothesis of physiological restrictions (particularly the tolerance to high temperatures)^3,5,14^, the results of this study are the first direct evidence to suggest that the thermal niche might be one of the main determinants of the ability of forest dung beetles to colonize open areas.

Specifically, we proposed predictions for the two main components of the thermal niche: endothermy and thermoregulation. Regarding endothermy, we expected (1) similar endothermy among species that are active during the colder periods of the day (E/N/C species) irrespectively of the preferred habitat (native forest, agroforestry parklands or open pastures) and (2) lower endothermy in species inhabiting habitats with extreme microclimatic conditions (open pastures without the shadow provided by trees) that are active during the hottest periods (diurnal species). Regarding physiological thermoregulation, we expected abdominal active heat transfer (AAHT) (to eliminate the excess of heat) in diurnal species irrespectively of the preferred habitat, and abdominal passive heat transfer (APHT) (to retain the heat) in E/N/C species.

Consistent with our prediction, open habitat diurnal species showed lower endothermy than species inhabiting forests, whereas E/N/C species showed similar endothermy in the three habitats. Canopy loss implies a drastic increase in ground temperature during the day but not during the night^2,5–7^. Consequently, diurnal species of open habitats should exhibit low endothermy to tolerate these extreme conditions or, in the case of species with high endothermy, an efficient active thermoregulation mechanism to remove excess heat to avoid the temperature of heat shock, between 42 and 45 °C during flight^28,37,38,68^. In our study, the two species that use open pastures during the day (*Canthon curvodilatus* and *C. podagricus*) were small and have very low endothermy, whereas species using this habitat during the evening/nocturnal/crepuscular periods were large and with higher endothermy.

The response of species that are active during the cold periods of the day (evening/nocturnal/crepuscular) was that expected under our prediction: similar endothermy independent of the habitat. This result is directly associated with the fact that temperatures in open and closed habitats during these periods were similar^2,5,6^. In general, all E/N/C species showed higher endothermy and body mass than diurnal species, probably as a consequence of the differences in temperature during the day and night (Fig. 1). The results obtained support the idea that, in dung beetles (and probably in most heterothermic insects), endothermy is an important physiological variable that could explain, at least partially, the drastic loss of species after forest conversion to open habitats and the ability of a reduced number of species to tolerate the new extreme conditions. Although these results are novel, two observations should be considered for future studies: (1) the inclusion of other variables associated with temperature (such as the start of heat stress temperature and heat regulation temperature described by Verdú et al.^69^) would help us to better explain the observed patterns, and (2) a larger number of individuals per species (mainly in the case of species that use open habitats) would standardize their populations to avoid biased results and greatly enrich the results of this type of study.

When considering the species response within habitats, the results of our study suggest that endothermy has an important role in species activity in habitats with high daily thermal amplitude. In our study, open habitats showed a thermal amplitude of 21 °C compared to 10–12 °C in closed habitats (amplitude was calculated as the difference between the maximum temperature during the day minus the minimum temperature during the night for the 12 day that the automatic temperature and humidity sensors were active, see Table S1). Although the thermal amplitude in tropical and subtropical habitats is less marked than in arid or semi-arid ecosystems, it seems to be enough to play an important role in defining the activity of species. Similar patterns have been described in other dung beetle assemblages. In semi-arid habitats of Huelva, Spain, for example, Verdú et al.^21^ showed that endothermy defined the activity of dung beetle species. Endothermy also plays an important role in altitudinal gradients^19^ and in the intra- and interspecific competitions of dung beetles^25,70^. However, to our knowledge, its role in the response of species to environmental disturbance by human activities has never been studied. Regarding body mass, the results suggest that this variable plays an important role in species activity in all habitats, not only in open habitats as is the case for endothermy, given that in all habitats diurnal species were smaller than E/N/C species.

Contrary to our predictions, diurnal species did not exhibit AAHT. We expected AAHT in diurnal species (mostly species with high endothermy) as a mechanism to tolerate high temperatures compared to E/N/C species; however, almost all diurnal species (with high and low endothermy) presented inefficient or undefined physiological thermoregulation mechanisms. Only *Coprophanaeus saphirinus* (which is diurnal) showed clearly APHT. For E/N/C species, we expected APHT; however, and like diurnal species, all species showed inefficient or undefined physiological thermoregulation mechanisms.

According to the results of this study, and the results of two previous studies performed in the same region^2,71^, the diurnal species with the highest endothermy, *Coprophanaeus saphirinus* (Fig. 5h), is active only in closed habitats (mainly native forests). This species may not be able to use open habitats, probably as a consequence of its high endothermy, although other factors such as the APHT or its thermal limits could play an important role. Individuals of this species can reach 8.5 °C above environmental temperature. Considering the extreme conditions of open habitats during the day (up to 35 °C), the high endothermy (7.2 °C in average) and heat retention, this species will probably quickly reach a thermal shock in open habitats (between 42 and 45 °C in insect flight)^28,37,38^. In contrast, the regulated microclimatic conditions in the understory of habitats preserving the tree canopy might be providing appropriate conditions during the day for this species in the Atlantic forest.

The remaining diurnal species studied exhibited medium or low endothermy and inefficient or undefined physiological thermoregulation mechanisms, in which the organisms increased their body temperature for take-off and then cool during flight (e.g. Fig. 5a–c, e, f). These species belong to the genus *Canthon* spp, which exhibit a distinctive flight behavior compared to other dung beetles within the subfamily Scarabaeinae^37,72,73^. Individuals fly with their elytra closed, liberating excess heat by regulating the separation between the elytra^28,73^ and the abdomen. Such type of flight, called *perching*, consists in a zig-zag flight to avoid the sun among leaves^72,73^. Desert ground beetles exhibit a similar behavior, alternating between the sun and the shade to maintain a constant body temperature^74,75^. According to this flight behavior, thermoregulation in this genus probably relies more on the behavior than on the physiology; the negative thorax and abdomen slopes during flight could be associated with the moment in which the individual does not bask the sun and remains in the shade. Like other insect taxa that present the same flight type (perching) (e.g. dragonflies, dipterans and butterflies), solar radiation represents the main resource of heat to increase thorax temperature during take-off^74,76^ and therefore the basking sun is necessary for take-off in these dung beetle species.

Two *Canthon* species used in this study were often captured in open habitats during the day; thus, the question is how these species maintain their flight in habitats without canopy cover? Both species are very small; small dung beetles tend to be thermoconformers (poikilotherms) during their flight^18,37^. In addition, they presented very low endothermy and generated heat for take-off (associated with their endothermy) but then their body temperature became dependent on the environmental temperature during the flight. Also, in the case of *Canthon podagricus*, some individuals showed AAHT, which suggests that, in some cases, they may also have the ability to remove excess heat in habitats with high temperatures.

The inefficient or undefined physiological thermoregulation mechanisms found in E/N/C species (e.g. Fig. 5i, l, m) could be associated with their flying behavior. Unlike *Canthon* species, species of the genus *Dichotomius* Hope (Giménez Gómez, personal observations) and *Deltochilum* Eschscholtz^28^ fly with their elytra open. In Scarabaeinae, open elytra in coordination with wing beating favor convective cooling^21,77^. Like *Canthon* spp., individuals increase body temperature for take-off (dependent on endothermy) but then, during the flight, they reduce their body temperature through convective cooling, explaining the negative slope of the thorax and abdomen temperatures observed during flight. In these cases, as well as in diurnal species, the thermoregulation mechanisms would be more behavioral than physiological.

In general, and based on the results of this study, we propose that dung beetle species in the Atlantic forest (and probably in other tropical and subtropical forests) show inefficient or undefined physiological thermoregulation mechanisms simply because the temperatures (mainly in the native forests) are stable and suitable for their survival. In species inhabiting the Atlantic forest, thermoregulation is probably more associated with behavioral strategies rather than with physiology.

Despite being important variables directly linked to insect flight, the rate of change in the thorax and abdomen temperatures during flight (slopes), the difference between both rates and the minimum thoracic tethered flapping temperature of species inhabiting closed and open habitats were similar, suggesting that these physiological variables have no influence on the ability of species to tolerate microclimatic changes. The results obtained for the rate of change in the thorax temperature during flight and the minimum thoracic tethered flapping temperature were surprising because both variables show a response similar to endothermy^36,38,52^; however, irrespective of endothermy, both variables were similar among species, suggesting that the direct relationship between endothermy, thorax temperature and minimum thoracic tethered flapping temperature is not a general rule.

Based on the results obtained, we can conclude that the ability or inability of dung beetles to increase their body temperature above environmental temperatures (excess temperature or endothermy) and the balance between heat gain and loss (thermoregulation) could explain, in part, the drastic loss of forest species after forest conversion in the Atlantic forest. Considering that endothermy differed among species belonging to the same genus (*Canthon*), and that it was lower only in species that use open habitats during the day, we suggest that endothermy is more related to habitat use than to phylogeny. Moreover, this is supported by previous studies showing that body temperature and thermoregulation tend to be independent of phylogenetic relationships among species^74^. In the case of diurnal species, only small species with very low endothermy (lower difference between body and environmental temperature) were able to use open habitats, whereas in the case of E/N/C species, we were not able to find a general rule. However, in general, E/N/C species found in open habitats were large and with high endothermy (greater difference between body and environmental temperatures).

We are aware of the limits of our study represented by the small number of tested individuals and species. However, these limitations were mainly associated with flight duration and behavior of the species. In some cases, reaching 40 s of continuous flight or pre-flight behavior was difficult and only a few individuals succeeded. However, as this is a multi-species physiological study, we believe that the number of individuals and the general consistency in the intra-specific physiological responses were enough to capture the principal responses. In relation to the number of species, the relatively small number of species considered in this study is a consequence of the number of indicator or detector species inhabiting each habitat. In open habitats, the number of species was lower than in other habitats because few species are able to tolerate the environmental conditions in open habitats. However, all typical species of open habitats were used in this study. Although our results were obtained in the southern Atlantic forest, a similar mechanism is probably occurring in other Neotropical forests since most studies have shown a similar reduction in dung beetle diversity after forest conversion (e.g. Audino et al.^78^, Beiroz et al.^79^). Also, previous studies showed the importance of other factors, such as soil type and vegetation structure^2,7,80^, influencing patterns of dung beetles diversity; however, these studies were not able to explain the ability (or inability) of species to exploit open human created habitats.

Physiological studies address not only the mechanisms of thermoregulation but also the thermal responses of individuals and the range of temperature required for growth and reproduction (thermal niche)^75^. In view of the accelerated process of forest and biodiversity loss and climate change, further profound physiological and ecological studies are essential to predict the response of species to environmental changes and to generate management recommendations to reduce the loss of diversity.

## Supplementary information

Supplementary Information.

## Data Availability

All data generated and analysed during this study are included in Supplementary Information.

## References

[1] Angilletta MJ (2009). Thermal Adaptation: A Theoretical and Empirical Analysis.

[2] Giménez Gómez VC, Verdú JR, Guerra Alonso CB, Zurita GA (2018). Relationship between land uses and diversity of dung beetles (Coleoptera: Scarabaeinae) in the southern Atlantic forest of Argentina: which are the key factors?. Biodivers. Conserv..

[3] Nichols E (2007). Global dung beetle response to tropical forest modification and fragmentation: a quantitative literature review and meta-analysis. Biol. Conserv..

[4] Barragán F, Moreno CE, Escobar F, Halffter G, Navarrete D (2011). Negative impacts of human land use on dung beetle functional diversity. PLoS ONE.

[5] Giménez Gómez VC, Verdú JR, Gómez-Cifuentes A, Vaz-de-Mello FZ, Zurita GA (2018). Influence of land use on the trophic niche overlap of dung beetles in the semideciduous Atlantic forest of Argentina. Insect Conserv. Divers..

[6] Gómez-Cifuentes A, Munevar A, Gimenez VC, Gatti MG, Zurita GA (2017). Influence of land use on the taxonomic and functional diversity of dung beetles (Coleoptera: Scarabaeinae) in the southern Atlantic forest of Argentina. J. Insect Conserv..

[7] Gómez-Cifuentes A, Giménez Gómez VC, Moreno C, Zurita GA (2019). Tree retention in cattle ranching systems partially preserves dung beetle diversity and functional groups in the semideciduous Atlantic forest. Basic Appl. Ecol..

[8] Halffter G, Arellano L (2002). Response of dung beetle diversity to human-induced changes in a tropical landscape. Biotropica.

[9] Gardner TA, Hernández MIM, Barlow J, Peres CA (2008). Understanding the biodiversity consequences of habitat change: the value of secondary and plantation forests for neotropical dung beetles. J. Appl. Ecol.

[10] Nichols E (2013). Trait-dependent response of dung beetle populations to tropical forest conversion at local and regional scales. Ecology.

[11] Sowig P (1995). Habitat selection and offspring survival rate in three paracoprid dung beetles: the influence of soil type and soil moisture. Ecography.

[12] Davis ALV, Van Aarde RJ, Scholtz CH, Delport JH (2002). Increasing representation of localized dung beetles across a chronosequence of regenerating vegetation and natural dune forest in South Africa. Glob. Ecol. Biogeogr..

[13] Almeida S, Louzada J, Sperber C, Barlow J (2011). Subtle land use change and tropical biodiversity: dung beetle communities in Cerrado grasslands and exotic pastures. Biotropica.

[14] Piccini I (2018). Dung beetles as drivers of ecosystem multifunctionality: are response and effect traits interwoven?. Sci. Total Environ..

[15] Di Bitetti MS, Placci G, Dietz LA (2003). A Biodiversity Vision for the Upper Paraná Atlantic Forest Ecoregion: Designing a Biodiversity Conservation Landscape and Setting Priorities for Conservation Action.

[16] Ribeiro MC, Metzger JP, Camargo Martensen A, Ponzoni FJ, Hirota MM (2009). The Brazilian Atlantic Forest: how much is left, and how is the remaining forest distributed? Implications for conservation. Biol. Conserv..

[17] Salomão RP, Lannuzzi L (2015). Dung beetle (Coleoptera, Scarabaeidae) assemblage of a highly fragmented landscape of Atlantic forest: from small to the largest fragments of northeastern Brazilian region. Rev. Bras. Entomol..

[18] Bartholomew GA, Heinrich B (1978). Endothermy in African dung beetles during flight, ball making, and ball rolling. J. Exp. Biol..

[19] Verdú JR, Arellano L, Numa C, Micó E (2007). Roles of endothermy in niche differentiation for ball-rolling dung beetles (Coleoptera: Scarabaeidae) along an altitudinal gradient. Ecol. Entomol..

[20] Caveney S, Scholtz CH, McIntyre P (1995). Patterns of daily flight activity in onitine dung beetles (Scarabaeinae: Onitini). Oecologia.

[21] Verdú JR, Díaz A, Galante E (2004). Thermoregulatory strategies in two closery related sympatric Scarabaeus species (Coleoptera: Scarabaeinae). Physiol. Entomol..

[22] Kingsolver JG (2009). The well-temperatured biologist. Am. Nat..

[23] Reis M (2011). A comparative study of the short term cold resistance response in distantly related Drosophila species: the role of regucalcin and frost. PLoS ONE.

[24] Harrison JF, Woods HA, Roberts SP (2012). Ecological and Environmental Physiology of Insects.

[25] Chown SL, Scholtz CH, Klok CJ, Jourbet FJ, Coles KS (1995). Ecophysiology, range contraction and survival of a geographically restricted African dung beetle (Coleoptera: Scarabaeidae). Funct. Ecol..

[26] Heath JE, Hanegan JL, Wilkin PJ, Heath MS (1971). Adaptation to the thermal responses of insects. Integr. Comp. Biol..

[27] Kristensen TN, Loeschcke V, Hoffmann AA (2007). Can artificially selected phenotypes influence a component of field fitness? Thermal selection and fly performance under thermal extremes. Proc. R. Soc. Lond. B Biol. Sci..

[28] Verdú JR, Lobo JM, Fattorini S (2008). Ecophysiology of thermorregulation in endothermic dung beetles: ecological and geographical implication. Insect Ecology and Conservation.

[29] Krogh A, Zeuthen E (1941). The mechanism of flight preparation in some insects. J. Exp. Biol..

[30] Heinrich B (1979). Thermoregulation of African and European honeybees during foraging, attack, and hive exits and returns. J. Exp. Biol..

[31] Verdú JR, Alba-Tercedor J, Jiménez-Manrique M (2012). Evidence of different thermoregulatory mechanisms between two sympatric Scarabaeus species using infrared thermography and microcomputer tomography. PLoS ONE.

[32] Chown SL, Terblanche JS (2006). Physiological diversity in insects: ecological and evolutionary contexts. Adv. Insect. Physiol..

[33] Terblanche JS, Deere JA, Clusells-Trullas S, Janion C, Chown SL (2007). Critical thermal limits depend on methodological context. Proc. R. Soc. Lond. B Biol. Sci..

[34] Vorhees AS, Gray EM, Bradley TJ (2013). Thermal resistance and performance correlate with climate in populations of a widespread mosquito. Physiol. Biochem. Zool..

[35] Gates DM (1980). Biophysical Ecology.

[36] Bartholomew GA, Casey TM (1977). Endothermy during terrestrial activity in large beetles. Science.

[37] Verdú JR, Arellano L, Numa C (2006). Thermoregulation in endotermic dung beetles (Coleoptera: Scarabaeidae): effect of body size and ecophysiological constraints in flight. J. Insect Physiol..

[38] Chown SL, Klok CJ, Simmons LW, Ridsdill-Smith TJ (2011). The ecological implications of physiological diversity in dung beetles. Ecology and Evolution of Dung Beetles.

[39] Oliveira-Filho AT, Fontes IAM (2000). Patterns of floristic differentiation among Atlantic forests in Southeastern Brazil and the influence of climate. Biotropica.

[40] Izquierdo AE, De Angelo CD, Aide TM (2008). Thirty years of human demography and land use change in the Atlantic Forest of Misiones, Argentina: an evaluation of the forest transition model. Ecol. Soc..

[41] Zurita GA, Bellocq MI (2012). Bird assemblages in anthropogenic habitats: identifying a suitability gradient for native species in the Atlantic forest. Biotropica.

[42] Cabrera AL (1971). Fitogeografía de Argentina. Boletín de sociedad Argentina de Botánica.

[43] Campanello PI, Montti L, Goldstein G, Mac Donagh P, Grossberg SP (2009). Reduced impact logging and post-harvesting forest management in the Atlantic Forest: alternative approaches to enhance canopy tree growth and regeneration and to reduce the impact of invasive species. Forest Management.

[44] Dufrene M, Legendre P (1997). Species assemblages and indicator species: the need for a flexible asymmetrical approach. Ecol. Monogr..

[45] McGeoch MA, Chown SL (1998). Scaling up the value of bioindicators. Trends Ecol. Evol..

[46] McGeoch MA, van Rensburg BJ, Botes A (2002). The verification and application of bioindicators: a case of study of dung beetles in a savanna ecosystem. J. Appl. Ecol..

[47] McCune, B. & Mefford, M. J. Multivariate Analysis of Ecological Data, Version 4.0. MjM Software, Gleneden Beach, Oregon, U.S.A. (1999).

[48] Hernández MIM (2002). The night and day of dung beetles (Coleoptera, Scarabaeidae) in the Serra do Japi, Brazil: elytra colour related to daily activity. Rev. Bras. Entomol..

[49] Hernández MIM, Monteiro LR, Favila ME (2011). The role of body size and shape in understanding competitive interactions within a community of neotropical dung beetles. J. Insect Sci..

[50] Heinrich B (1993). Hot-blooded Insects: Strategies and Mechanisms of Thermoregulation.

[51] Vannier G (1994). The thermobiological limits of some freezing intolerant insects: the supercooling and thermostupor points. Acta Oecol..

[52] Chown SL, Nicolson SW (2004). Insect Physiological Ecology: Mechanisms and Patterns.

[53] Gallego B, Verdú JR, Carrascal LM, Lobo JM (2016). A protocol for analyzing thermal stress in insects using infrared thermography. J. Therm. Biol..

[54] Merrick M (2004). Temperature regulation in burying beetles (*Nicrophorus* spp.: Coleoptera: Silphidae): effects of body size, morphology and environmental temperature. J. Exp. Biol..

[55] Tyndale-Biscoe M (1984). Age-grading methods in adult insects: a review. Bull. Entomol. Res..

[56] Verdú JR, Casa JL, Lobo JM, Numa C (2010). Dung beetles eat acorns to increase their ovarian development and thermal tolerance. PLoS ONE.

[57] StatsDirect Ltd StatsDirect Statistical Software, StatsDirect, U.K.

[58] May ML (1976). Thermoregulation and adaptation to temperature in dragonflies (Odonata: Anisoptera). Ecol. Monogr..

[59] Fox, J. & Weisberg, S. An {R} Companion to Applied Regression, Second Edition. Thousand Oaks CA: Sage. https://socserv.socsci.mcmaster.ca/jfox/Books/Companion (2011).

[60] Bates D, Maechler M, Bolker B, Walker S (2015). Fitting linear mixed-effects models using lme4. J. Stat. Softw..

[61] Length, R. Emmeans: Estimated Marginal Means, aka Least-Squares Means. R package version 1.2.3. https://CRAN.R-project.org/package=emmeans (2018).

[62] Dinno, A. Conover.test: conover-iman test of multiple comparisons using rank sums. R package version 1.1.4. https://CRAN.R-project.org/package=conover.test (2017).

[63] Di Rienzo, J. A. *et al*. W. InfoStat version 3241 2016. Grupo InfoStat, FCA, Universidad Nacional de Córdoba, Argentina (2016).

[64] Moran DM (2013). Arguments for rejecting the sequential Bonferroni in ecological studies. Oikos.

[65] Campos RC, Hernández MIM (2015). The importance of maize management on dung beetle communities in Atlantic forest fragment. PLoS ONE.

[66] Filgueiras BKC, Tabarelli M, Leal I, Vaz-De-Mello FZ, Iannuzzi L (2015). Dung beetle persistence in human-modified landscapes: combining indicator species with anthropogenic land uses and fragmentation- related effects. Ecol. Indic..

[67] Tavares A (2019). Eucalyptus plantations as hybrid ecosystems: implications for species conservation in the Brazilian Atlantic forest. For. Ecol. Manag..

[68] Smolka J (2012). Dung beetles use their dung ball as a mobile thermal refuge. Curr. Biol..

[69] Verdú JR, Cortez V, Oliva D, Giménez-Gómez V (2019). Thermoregulatory syndromes of two sympatric dung beetles with low energy costs. J. Insect Physiol..

[70] Heinrich B, Bartholomew GA (1979). Roles of endothermy and size in inter- and intraspecific competition for elephant dung in an African dung beetle, *Scarabaeus laevistriatus*. Physiol. Zool..

[71] Da Silva PG, Hernández MIM (2016). Spatial variation of dung beetle assemblages associated with forest structure in remnants of southern Brazilian Atlantic Forest. Rev. Bras. Entomol..

[72] May ML (1979). Insect thermoregulation. Annu. Rev. Entomol..

[73] Young OP (1984). Perching of neotropical dung beetles on leaf surfaces: an example of behavioral thermoregulation?. Biotropica.

[74] Heinrich B (1995). Insect thermoregulation. Endeavour.

[75] Edney EB (1971). Body temperatures of tenebrionid beetles in the Namib Desert of Southern Africa. J. Exp. Biol..

[76] Casey TM (1988). Thermoregulation and heat exchange. Adv. Insect Physiol..

[77] Halffter G, Matthews EG (1966). The natural history of dung beetles of the subfamily Scarabaeinae (Coleoptera: Scarabaeidae). Soc. Mex. Entomol..

[78] Audino LD, Louzada J, Comita L (2014). Dung beetles as indicators of tropical forest restoration success: is it possible to recover species and functional diversity?. Biol. Conserv..

[79] Beiroz W (2018). Spatial and temporal shifts in functional and taxonomic diversity of dung beetles in a human-modified tropical forest landscape. Ecol. Indic..

[80] Gómez-Cifuentes A, Vespa N, Semmanrtín M, Zurita GA (2020). Canopy cover is a key factor to preserve the ecological functions of dung beetles in the southern Atlantic Forest. Appl. Soil Ecol..

