# Supplementary material for: Thermal niche helps to explain the ability of dung beetles to exploit disturbed habitats
Source: Sci Rep. 2020 Aug 7;10:13364. doi: 10.1038/s41598-020-70284-8 (PMC7414905; doi:10.1038/s41598-020-70284-8)
Supplement: Supplementary file 1 — Supplementary Information. [file 41598_2020_70284_MOESM1_ESM.pdf]

Journal: Scientific Reports

Thermal niche helps to explain the ability of dung beetles to exploit disturbed habitats

Victoria C. Giménez Gómez[1*], José R. Verdú[2] and Gustavo A. Zurita[1,3]

1. Instituto de Biología Subtropical, Universidad Nacional de Misiones-CONICET Puerto Iguazú, Misiones, Argentina.

2. Instituto Universitario de Investigación – Centro Iberoamericano de la Biodiversidad, Universidad de Alicante, Spain.

3. Facultad de Ciencias Forestales, Universidad Nacional de Misiones-CONICET, Eldorado, Misiones, Argentina.

*Correspondence author: V.C. Giménez Gómez. Instituto de Biología Subtropical- Universidad Nacional de Misiones – CONICET, Bertoni 85, Puerto Iguazú, Misiones (3370), Argentina. Tel: 54-3757- 423511. E-mail: vc_gimenezgomez@hotmail.com. ORCID 0000-0002-8330-977X.

Supplementary Information

Tables

Table S1 Mean, maximum and minimum temperatures during the day and night, considering 12 continuous days, in: native forests, agroforestry parklands and open pastures of the Atlantic forest of Argentina.

		**Native forests (°C)**	**Agroforestry parklands (°C)**	**Open pastures (°C)**
**Day**	Mean	22.6	24.1	30.2
	Maximum	24.6	27.6	34.9
	Minimum	17.0	17.5	21.0
**Night**	Mean	17.4	17.6	16.0
	Maximum	20.7	21.5	19.9
	Minimum	15.4	15.5	13.6

Table S2 Species selected according to the Indicator Value Method (IndVal). When the IndVal index value was ≥ 70 and the P value < 0.05, the species was considered as an "indicator species (IS)", whereas when the IndVal index value was < 70 and the P value < 0.05, the species was considered as a "detector species (DS)".

Species	***IndVal index value***	***P value***	***Habitat***	***Species type***
Canthon conformis	97.7	0.0010	Agroforestry parklands	IS
Canthon curvodilatus	80	0.0150	Open pastures	IS
Canthon histrio	99.8	0.0020	Agroforestry parklands	IS
Canthon podagricus	100	0.0030	Open pastures	IS
Canthon quinquemaculatus	87.4	0.00020	Agroforestry parklands	IS
Coprophanaeus cyanescens	60	0.0330	Agroforestry parklands	DS
Coprophanaeus saphirinus	64.5	0.049	Native forests	DS
Deltochilum brasiliensis	100	0.0030	Native forests	IS
Deltochilum furcatum	80.6	0.0110	Native forests	IS
Deltochilum aff. *komareki*	84.3	0.0020	Agroforestry parklands	IS
Dichotomius carbonarius	84.9	0.0070	Agroforestry parklands	IS
Dichotomius mormon	66.7	0.0190	Agroforestry parklands	DS
Dichotomius nisus	100	0.0030	Open pastures	IS
Dichotomius sericeus	63.7	0.0250	Agroforestry parklands	DS

Table S3 Emissivity value of the cuticle of each species at different temperatures (50-80 °C), determined using fresh cuticle. To take the measurements, black insulating tape, whose emissivity is known (ε = 0.95), was used as a reference, following the protocol of Verdú *et al.*[31] and Gallego *et al.*[53]. The last column shows the average emissivity used for correction for each species.

SPECIES	50 °C	60 °C	70 °C	80 °C	AVERAGE
Canthon conformis	0.95	0.94	0.95	0.94	0.95
Canthon curvodilatus	0.95	0.94	0.95	0.94	0.95
Canthon histrio	0.94	0.94	0.94	0.95	0.94
Canthon podagricus	0.95	0.94	0.95	0.94	0.95
Canthon quinquemaculatus	0.94	0.94	0.94	0.95	0.94
Canthon smaragdulus	0.95	0.95	0.94	0.94	0.95
Coprophanaeus cyanescens	0.93	0.93	0.92	0.92	0.93
Coprophanaeus saphirinus	0.94	0.94	0.95	0.94	0.94
Deltochilum brasiliensis	0.93	0.94	0.94	0.94	0.94
Deltochilum furcatum	0.89	0.89	0.89	0.91	0.90
Deltochilum aff. *komareki*	0.93	0.94	0.94	0.94	0.94
Deltochilum morbillosum	0.93	0.94	0.94	0.94	0.94
Dichotomius carbonarius	0.90	0.89	0.89	0.90	0.90
Dichotomius mormon	0.93	0.93	0.92	0.91	0.92
Dichotomius nisus	0.93	0.93	0.92	0.91	0.92
Dichotomius sericeus	0.92	0.93	0.93	0.92	0.93
Ontherus sulcator	0.93	0.94	0.93	0.93	0.93

Table S4 Results of the daily activity of dung beetles in the native forest, agroforestry parklands and open pastures in the Atlantic forest of Argentina. **P1:** diurnal period and **P2:** evening/nocturnal/crepuscular periods. The values highlighted in bold represent the percentages ≥ 60 used to identify the activity of dung beetle species.

Species	***Native forests***					***Agroforestry parklands***					***Open pastures***				
	**P1**	**(%)**	**P2**	**(%)**	**TOTAL**	**P1**	**(%)**	**P2**	**(%)**	**TOTAL**	**P1**	**(%)**	**P2**	**(%)**	**TOTAL**
Canthidium sp.	4	**80**	1	20	5	0	0	0	0	0	0	0	0	0	0
Canthon conformis	5	**100**	0	0	5	72	**90**	8	10	80	0	0	0	0	0
Canthon histrio	0	0	0	0	0	6	**100**	0	0	6	1	**100**	0	0	1
Canthon podagricus	0	0	0	0	0	0	0	0	0	0	9	**60**	6	40	15
Canthon quinquemaculatus	349	**99**	2	1	351	301	**86**	47	14	348	1	**100**	0	0	1
Chalcocopris hesperus	7	**100**	0	0	7	0	0	0	0	0	0	0	0	0	0
Coprophanaeus cyanescens	0	0	6	**100**	6	0	0	1	**100**	1	0	0	0	0	0
Coprophanaeus saphirinus	42	**70**	18	30	60	1	50	1	50	2	0	0	0	0	0
Deltochilum brasiliense	1	**100**	0	0	1	0	0	0	0	0	0	0	0	0	0
Deltochilum furcatum	0	0	9	**100**	9	0	0	2	**100**	2	0	0	0	0	0
Deltochilum aff. *komareki*	1	3	31	**97**	32	3	12	21	**88**	24	0	0	0	0	0
Deltochilum morbillosum	1	50	1	50	2	0	0	0	0	0	0	0	0	0	0
Dichotomius mormon	0	0	2	**100**	2	0	0	0	0	0	0	0	0	0	0
Dichotomius nisus	0	0	0	0	0	0	0	0	0	0	0	0	9	**100**	9
Dichotomius sericeus	12	18	54	**82**	66	6	14	37	**86**	43	0	0	0	0	0
Eurysternus caribaeus	6	50	6	50	12	0	0	1	**100**	1	0	0	0	0	0
Eurysternus parallelus	115	**92**	10	8	125	21	**100**	0	0	21	1	**100**	0	0	1
Ontherus sulcator	0	0	0	0	0	0	0	0	0	0	0	0	3	**100**	3
Onthophagus catharinensis	17	17	85	83	102	0	0	0	0	0	0	0	0	0	0
Onthophagus tristis	1	**100**	0	0	1	0	0	0	0	0	0	0	0	0	0
Scybalocanthon nigriceps	3	**100**	0	0	3	0	0	0	0	0	0	0	0	0	0
Uroxys dilaticollis	0	0	1	**100**	1	0	0	1	**100**	1	0	0	0	0	0

Figures

Figure S1 Study area in the southern Atlantic forest of Argentina, showing the land uses sampled. Dots: native forests (NF), squares: agroforestry parklands (AP) and crosses: open pastures (OP). ArcGIS software v10.7 under an Esri Conservation Program license was used to create the map. Copyright © Esri.

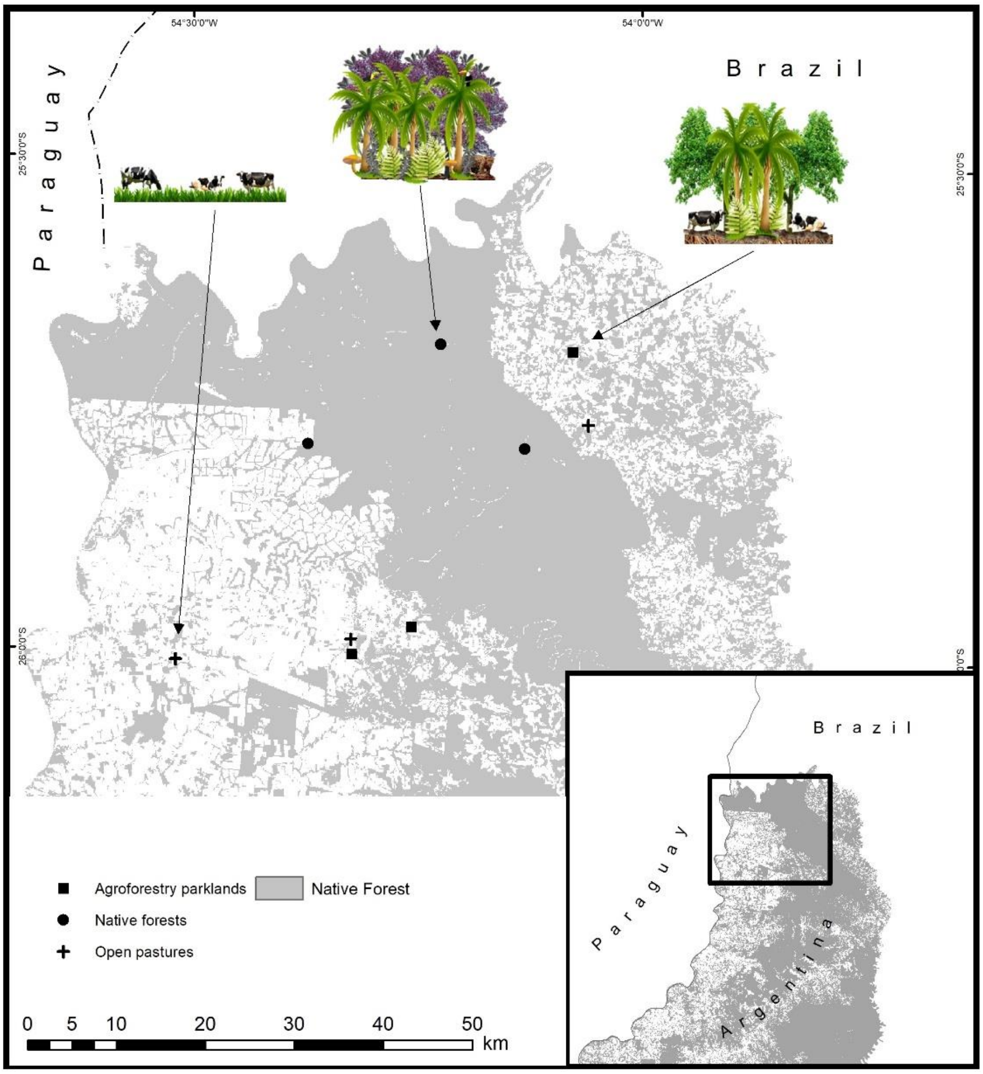

Datasets

Dataset on which the analyses were performed. T_{th} min of take-off = minimum thoracic take-off temperature, sT_{th} = thorax slope, sT_{abd} = abdomen slope, sT_{env} = environment temperature, sT_{th}-sT_{abd} = difference between sT_{th} and sT_{abd}.

Species	Habitat	Activity	Mass	Endothermy	T_{th} min of take-off	sT_{th}	sT_{abd}	sT_{env}	sT_{th}-sT_{abd}
Canthon conformis	Agroforestry parkland	Diurnal	0.069	1.876	28.839	-0.0322	-0.0251	-0.0018	-0.0071
Canthon conformis	Agroforestry parkland	Diurnal	0.059	3.229	30.034	0.0132	-0.0315	0.0018	0.0447
Canthon conformis	Agroforestry parkland	Diurnal	0.03	1.269	27.054	-0.0044	-0.0039	-0.0011	-0.0005
Canthon conformis	Agroforestry parkland	Diurnal	0.04	4.532	27.95	-0.0166	-0.018	0.0009	0.0014
Canthon conformis	Agroforestry parkland	Diurnal	0.043	3.441	26.002	0.0037	0.0011	0.0006	0.0026
Canthon conformis	Agroforestry parkland	Diurnal	0.03	3.429	26.007	0.0061	0.0049	0.004	0.0012
Canthon conformis	Agroforestry parkland	Diurnal	0.032	3.411	27.574	-0.0092	-0.009	0.0001	-0.0002
Canthon conformis	Agroforestry parkland	Diurnal	0.037	3.241	25.721	0.0066	0.0035	0.0034	0.0031
Canthon conformis	Agroforestry parkland	Diurnal	0.033	3.265	26.807	0.0007	0.0013	0.001	-0.0006
Canthon conformis	Agroforestry parkland	Diurnal	0.038	4.023	28.39	-0.0159	-0.0159	0.0003	0
Canthon conformis	Agroforestry parkland	Diurnal	0.035	3.347	27.185	-0.0023	-0.0032	0.0004	0.0009
Canthon conformis	Agroforestry parkland	Diurnal	0.039	3.174	27.046	0.0015	0.002	0.0009	-0.0005
Canthon conformis	Agroforestry parkland	Diurnal	0.043	5.074	29.703	-0.0261	-0.0194	-0.0002	-0.0067
Canthon curvodilatus	Open pasture	Diurnal	0.025	0.383	27.444	0.0034	0.0038	-0.0006	-0.0004
Canthon curvodilatus	Open pasture	Diurnal	0.024	0.418	27.99	0.0024	0.0028	0.0007	-0.0004
Canthon curvodilatus	Open pasture	Diurnal	0.03	0.402	28.03	-0.0003	0.0016	0.0013	-0.0019
Canthon curvodilatus	Open pasture	Diurnal	0.029	0.687	29.229	-0.011	-0.0097	0.0001	-0.0013
Canthon histrio	Agroforestry parkland	Diurnal	0.132	4.677	30.621	-0.0007	0.0034	-0.0001	-0.0041
Canthon histrio	Agroforestry parkland	Diurnal	0.141	4.19	30.829	-0.021	-0.008	0.00008	-0.013
Canthon histrio	Agroforestry parkland	Diurnal	0.113	3.644	30.247	-0.0074	-0.0044	-0.0004	-0.003
Canthon histrio	Agroforestry parkland	Diurnal	0.139	4.431	29.853	0.0075	0.0137	-0.0007	-0.0062
Canthon histrio	Agroforestry parkland	Diurnal	0.162	3.842	29.928	-0.0204	0.01	0.0022	-0.0304
Canthon histrio	Agroforestry parkland	Diurnal	0.176	3.76	29.512	-0.0054	0.0026	-0.0043	-0.008

Canthon histrio	Agroforestry parkland	Diurnal	0.1818	2.892	30.785	0.0031	0.0041	0.0005	-0.001
Canthon histrio	Agroforestry parkland	Diurnal	0.1283	2.729	30.023	0.0039	0.0056	0.0028	-0.0017
Canthon podagricus	Open pasture	Diurnal	0.026	0.521	28.248	0.0029	0.0015	0.001	0.0014
Canthon podagricus	Open pasture	Diurnal	0.032	0.643	28.19	0.0021	0.0021	-0.0013	0
Canthon podagricus	Open pasture	Diurnal	0.023	2.994	27.674	0.0254	0.048	0.002	-0.0226
Canthon podagricus	Open pasture	Diurnal	0.025	1.462	28.019	0.0029	0.0015	0.0022	0.0014
Canthon podagricus	Open pasture	Diurnal	0.032	1.333	27.108	-0.0181	-0.0188	-0.0013	0.0007
Canthon podagricus	Open pasture	Diurnal	0.026	0.664	27.62	-0.004	-0.003	-0.0008	-0.001
Canthon podagricus	Open pasture	Diurnal	0.023	0.696	27.686	-0.0144	-0.0175	0.0018	0.0031
Canthon podagricus	Open pasture	Diurnal	0.028	0.761	27.321	-0.0008	-0.0007	-0.0011	-0.0001
Canthon podagricus	Open pasture	Diurnal	0.03	0.54	27.255	-0.0079	-0.0136	-0.0015	0.0057
Canthon podagricus	Open pasture	Diurnal	0.031	1.424	28.382	0.0281	0.0278	-0.0006	0.0003
Canthon podagricus	Open pasture	Diurnal	0.031	1.522	28.38	-0.0057	-0.0071	0.0008	0.0014
Canthon quinquemaculatus	Agroforestry parkland	Diurnal	0.14	1.572	29.486	-0.0049	-0.005	0.0003	0.0001
Canthon quinquemaculatus	Agroforestry parkland	Diurnal	0.17	1.549	28.505	-0.0101	-0.0108	-0.0012	0.0007
Canthon quinquemaculatus	Agroforestry parkland	Diurnal	0.18	1.795	28.633	-0.0024	-0.0014	-0.0005	-0.001
Canthon quinquemaculatus	Agroforestry parkland	Diurnal	0.19	1.5	28.55	-0.0083	-0.0067	-0.0008	-0.0016
Canthon quinquemaculatus	Agroforestry parkland	Diurnal	0.19	2.466	29.946	-0.007	-0.0051	0.00006	-0.0019
Canthon quinquemaculatus	Agroforestry parkland	Diurnal	0.16	2.221	30.955	-0.0053	-0.0035	-0.00007	-0.0018
Canthon quinquemaculatus	Agroforestry parkland	Diurnal	0.19	1.871	28.687	-0.0051	0.0002	0.0002	-0.0053
Canthon quinquemaculatus	Agroforestry parkland	Diurnal	0.13	1.811	28.113	-0.0108	0.0021	-0.0004	-0.0129
Canthon quinquemaculatus	Agroforestry parkland	Diurnal	0.16	1.162	27.822	0.0004	0.0014	-0.0002	-0.001
Canthon quinquemaculatus	Agroforestry parkland	Diurnal	0.192	2.467	30.037	-0.0093	-0.0074	0.0014	-0.0019
Canthon smaragdulus	Native forest	Diurnal	0.272	2.186	26.32	-0.0245	-0.0143	0.0008	-0.0102
Canthon smaragdulus	Native forest	Diurnal	0.185	3.29	27.997	-0.022	-0.0235	-0.0003	0.0015
Canthon smaragdulus	Native forest	Diurnal	0.28	2.194	27.89	-0.0179	-0.0147	-0.0002	-0.0032
Canthon smaragdulus	Native forest	Diurnal	0.088	3.077	28.5	0.0039	0.00143	0.0035	0.00247
Canthon smaragdulus	Native forest	Diurnal	0.102	3.148	28.714	0.0362	0.0174	-0.0008	0.0188
Canthon smaragdulus	Native forest	Diurnal	0.118	3.275	29.033	-0.0022	0.0013	-0.001	-0.0035

Canthon smaragdulus	Native forest	Diurnal	0.102	3.911	28.769	0.0043	0.0024	-0.001	0.0019
Canthon smaragdulus	Native forest	Diurnal	0.098	4.026	30.767	0.0024	0.0028	0.0023	-0.0004
Coprophanaeus cyanescens	Agroforestry parkland	Evening/nocturnal/crepuscular	2.14	6.678	33.373	0.0265	0.0745	0.002	-0.048
Coprophanaeus cyanescens	Agroforestry parkland	Evening/nocturnal/crepuscular	1.8	5.215	33.508	0.0003	0.0004	-0.0008	-0.0001
Coprophanaeus cyanescens	Agroforestry parkland	Evening/nocturnal/crepuscular	1.63	5.12	33.62	0.0053	0.0004	0.002	0.0049
Coprophanaeus cyanescens	Agroforestry parkland	Evening/nocturnal/crepuscular	1.76	12.605	34.279	-0.0063	0.014	0.003	-0.0203
Coprophanaeus cyanescens	Agroforestry parkland	Evening/nocturnal/crepuscular	1.28	11.744	34.451	-0.006	-0.0153	0.0047	0.0093
Coprophanaeus cyanescens	Agroforestry parkland	Evening/nocturnal/crepuscular	1.67	13.939	36.017	0.0089	0.0022	-0.0007	0.0067
Coprophanaeus saphirinus	Native forest	Diurnal	1.17	5.918	30.647	0.0004	0.001	-0.0009	-0.0006
Coprophanaeus saphirinus	Native forest	Diurnal	0.307	8.433	31.539	0.0089	-0.0181	-0.0007	0.027
Coprophanaeus saphirinus	Native forest	Diurnal	0.253	8.211	33.542	0.0034	-0.0117	0.0065	0.0151
Coprophanaeus saphirinus	Native forest	Diurnal	0.387	6.79	32.466	0.014	-0.0094	-0.0005	0.0234
Coprophanaeus saphirinus	Native forest	Diurnal	0.356	5.225	30.313	0.0064	0.0029	0.0021	0.0035
Coprophanaeus saphirinus	Native forest	Diurnal	0.254	7.096	30.527	0.0013	-0.0003	0.0047	0.0016
Coprophanaeus saphirinus	Native forest	Diurnal	0.273	7.803	30.652	0.0204	0.0007	0.0052	0.0197
Coprophanaeus saphirinus	Native forest	Diurnal	0.267	8.312	34.905	0.0035	-0.0054	-0.004	0.0089
Coprophanaeus saphirinus	Native forest	Diurnal	0.372	8.472	35.393	-0.0067	-0.0181	-0.00003	0.0114
Coprophanaeus saphirinus	Native forest	Diurnal	0.453	7.113	30.377	0.0118	0.0062	0.0014	0.0056
Coprophanaeus saphirinus	Native forest	Diurnal	0.422	8.427	31.033	-0.0015	0.007	0.0023	-0.0085
Coprophanaeus saphirinus	Native forest	Diurnal	0.38	6.067	29.104	-0.0236	-0.0069	0.0012	-0.0167
Coprophanaeus saphirinus	Native forest	Diurnal	0.59	6.978	31.292	-0.0017	-0.0037	0.0005	0.002
Coprophanaeus saphirinus	Native forest	Diurnal	0.44	6.713	34.682	0.00006	-0.0141	0.0001	0.01416
Deltochilum brasiliensis	Native forest	Evening/nocturnal/crepuscular	1.026	4.804	32.271	-0.0125	-0.0132	-0.0008	0.0007
Deltochilum brasiliensis	Native forest	Evening/nocturnal/crepuscular	1.126	3.226	29.742	-0.012	0.0131	-0.0013	-0.0251
Deltochilum brasiliensis	Native forest	Evening/nocturnal/crepuscular	0.954	3.071	29.668	0.0015	-0.0042	0.0001	0.0057
Deltochilum brasiliensis	Native forest	Evening/nocturnal/crepuscular	0.879	1.948	28.401	-0.0002	-0.0011	-0.0005	0.0009
Deltochilum brasiliensis	Native forest	Evening/nocturnal/crepuscular	1.062	1.189	31.357	-0.0001	0.0016	0.0002	-0.0017
Deltochilum brasiliensis	Native forest	Evening/nocturnal/crepuscular	1.023	1.194	31.386	-0.0011	-0.0011	-0.0001	0
Deltochilum brasiliensis	Native forest	Evening/nocturnal/crepuscular	0.998	1.635	31.274	-0.00007	-0.0019	-0.0021	0.00183

Deltochilum brasiliensis	Native forest	Evening/nocturnal/crepuscular	0.994	1.632	31.343	0.003	-0.00006	0.0006	0.00306
Deltochilum furcatum	Native forest	Evening/nocturnal/crepuscular	0.6	3.07	28.329	0.0249	0.0045	0.0002	0.0204
Deltochilum furcatum	Native forest	Evening/nocturnal/crepuscular	0.7	4.426	30.573	-0.0009	-0.01	0.002	0.0091
Deltochilum furcatum	Native forest	Evening/nocturnal/crepuscular	0.689	4.46	29.915	0.0071	-0.0011	0.0018	0.0082
Deltochilum aff.*komareki*	Agroforestry parkland	Evening/nocturnal/crepuscular	0.239	3.507	29.112	0.0009	-0.007	-0.0022	0.0079
Deltochilum aff.*komareki*	Agroforestry parkland	Evening/nocturnal/crepuscular	0.202	2.192	29.885	-0.0001	-0.0006	0.00006	0.0005
Deltochilum aff.*komareki*	Agroforestry parkland	Evening/nocturnal/crepuscular	0.288	1.129	28.006	-0.0014	0.0004	-0.0003	-0.0018
Deltochilum aff.*komareki*	Agroforestry parkland	Evening/nocturnal/crepuscular	0.272	1.429	28.08	0.0166	0.0014	0.0029	0.0152
Deltochilum aff.*komareki*	Agroforestry parkland	Evening/nocturnal/crepuscular	0.16	1.837	29.001	-0.0057	-0.0043	0.0026	-0.0014
Deltochilum morbillosum	Native forest	Evening/nocturnal/crepuscular	0.206	2.801	29.309	0.0002	-0.0019	0.0002	0.0021
Deltochilum morbillosum	Native forest	Evening/nocturnal/crepuscular	0.235	3.025	30.654	-0.0026	-0.0017	0.0001	-0.0009
Deltochilum morbillosum	Native forest	Evening/nocturnal/crepuscular	0.222	2.169	28.695	-0.001	0.0003	0.0002	-0.0013
Deltochilum morbillosum	Native forest	Evening/nocturnal/crepuscular	0.223	2.367	32.447	0.0054	-0.0012	-0.0015	0.0066
Deltochilum morbillosum	Native forest	Evening/nocturnal/crepuscular	0.271	2.433	32.793	0.0019	0.0021	0.0003	-0.0002
Deltochilum morbillosum	Native forest	Evening/nocturnal/crepuscular	0.209	3.413	33.574	-0.0029	-0.0011	0.0005	-0.0018
Dichotomius carbonarius	Agroforestry parkland	Evening/nocturnal/crepuscular	0.578	4.634	31.91	-0.0046	0.0022	0.0013	-0.0068
Dichotomius carbonarius	Agroforestry parkland	Evening/nocturnal/crepuscular	0.476	4.154	31.56	0.0003	-0.0012	0.0005	0.0015
Dichotomius carbonarius	Agroforestry parkland	Evening/nocturnal/crepuscular	0.523	3.724	30.894	-0.0053	0.0004	-0.0033	-0.0057
Dichotomius carbonarius	Agroforestry parkland	Evening/nocturnal/crepuscular	0.548	4.132	29.837	-0.0015	-0.0028	-0.0012	0.0013
Dichotomius carbonarius	Agroforestry parkland	Evening/nocturnal/crepuscular	0.523	3.107	29.381	-0.0007	0.0011	0.047	-0.0018
Dichotomius carbonarius	Agroforestry parkland	Evening/nocturnal/crepuscular	0.438	5.081	31.163	-0.0092	-0.0052	0.0018	-0.004
Dichotomius carbonarius	Agroforestry parkland	Evening/nocturnal/crepuscular	0.589	3.461	29.916	-0.0024	-0.0024	0.0011	0
Dichotomius mormon	Agroforestry parkland	Evening/nocturnal/crepuscular	0.989	6.29	33.005	-0.001	-0.0004	0.00005	-0.0006
Dichotomius mormon	Agroforestry parkland	Evening/nocturnal/crepuscular	1.157	6.64	33.207	-0.0042	-0.0022	0.0001	-0.002
Dichotomius mormon	Agroforestry parkland	Evening/nocturnal/crepuscular	0.709	7.57	32.64	-0.0063	-0.0054	0.0004	-0.0009
Dichotomius mormon	Agroforestry parkland	Evening/nocturnal/crepuscular	1.012	3.339	29.497	0.0116	0.01	0.0009	0.0016
Dichotomius mormon	Agroforestry parkland	Evening/nocturnal/crepuscular	1.182	4.163	30.76	0.0001	0.0015	-0.0013	-0.0014
Dichotomius mormon	Agroforestry parkland	Evening/nocturnal/crepuscular	1.106	5.367	32.109	-0.0005	0.0002	-0.0004	-0.0007
Dichotomius mormon	Agroforestry parkland	Evening/nocturnal/crepuscular	0.905	3.216	30.242	0.0025	0.0056	0.0017	-0.0031

Dichotomius mormon	Agroforestry parkland	Evening/nocturnal/crepuscular	1.112	3.617	30.841	0.0004	0.0031	0.0016	-0.0027
Dichotomius nisus	Open pasture	Evening/nocturnal/crepuscular	0.528	4.273	27.268	0.0004	-0.0009	-0.0005	0.0013
Dichotomius nisus	Open pasture	Evening/nocturnal/crepuscular	0.498	6.075	29.002	0.0025	0.0004	0.0015	0.0021
Dichotomius nisus	Open pasture	Evening/nocturnal/crepuscular	0.524	7.204	31.939	0.0008	0.0023	0.0049	-0.0015
Dichotomius nisus	Open pasture	Evening/nocturnal/crepuscular	0.499	7.306	31.959	-0.0016	0.0006	0.0025	-0.0022
Dichotomius nisus	Open pasture	Evening/nocturnal/crepuscular	0.532	5.507	30.292	-0.0009	-0.0032	0.0006	0.0023
Dichotomius nisus	Open pasture	Evening/nocturnal/crepuscular	0.485	6.495	30.907	0.0022	0.0077	-0.0005	-0.0055
Dichotomius sericeus	Agroforestry parkland	Evening/nocturnal/crepuscular	0.359	2.456	29.413	-0.0023	0.0009	-0.0017	-0.0032
Dichotomius sericeus	Agroforestry parkland	Evening/nocturnal/crepuscular	0.372	3.569	30.017	0.047	0.0105	-0.0011	0.0365
Dichotomius sericeus	Agroforestry parkland	Evening/nocturnal/crepuscular	0.359	4.005	30.133	0.006	-0.0046	-0.0009	0.0106
Dichotomius sericeus	Agroforestry parkland	Evening/nocturnal/crepuscular	0.396	2.865	28.697	-0.0011	0.0032	-0.0003	-0.0043
Dichotomius sericeus	Agroforestry parkland	Evening/nocturnal/crepuscular	0.196	1.003	27.406	-0.00004	0.0011	0.0006	-0.00114
Ontherus sulcator	Open pasture	Evening/nocturnal/crepuscular	0.316	1.978	30.649	0.0078	0.0086	0.0016	-0.0008
Ontherus sulcator	Open pasture	Evening/nocturnal/crepuscular	0.325	1.862	32.198	0.0076	0.007	0.0003	0.0006
Ontherus sulcator	Open pasture	Evening/nocturnal/crepuscular	0.298	2.614	33.628	-0.0032	-0.0024	0.0002	-0.0008
Ontherus sulcator	Open pasture	Evening/nocturnal/crepuscular	0.302	2.641	32.793	-0.0003	-0.0004	-0.0002	0.0001
Ontherus sulcator	Open pasture	Evening/nocturnal/crepuscular	0.343	2.324	32.267	0.0003	0.0017	0.0002	-0.0014
Ontherus sulcator	Open pasture	Evening/nocturnal/crepuscular	0.323	2.074	32.346	-0.00003	-0.0027	-0.0037	0.00267
